# MUC1 drives ferroptosis resistance in ICC via Src‐mediated FSP1 deubiquitination and myristoylation

**DOI:** 10.1002/ctm2.70495

**Published:** 2025-10-09

**Authors:** Yuqiao Zhao, Shifeng Yang, Lei Huang, Xuyun Liu, Qixiang Han, Qichao Niu, Siyi Li, Chuanlie Zhou, Boshi Sun, Yanmei Yang, Xinyu Zhang

**Affiliations:** ^1^ Department of General Surgery The 2nd Affiliated Hospital of Harbin Medical University Harbin China; ^2^ Center for Endemic Disease Control Chinese Center for Disease Control and Prevention Harbin Medical University Harbin China

**Keywords:** ferroptosis, intrahepatic cholangiocarcinoma, MUC1, post‐translational modification

## Abstract

**Background:**

Intrahepatic cholangiocarcinoma (ICC) exhibits poor prognosis and limited therapeutic options. Ferroptosis represents a promising therapeutic strategy, yet resistance mechanisms remain poorly understood. This study investigated the role of mucin 1 (MUC1) in regulating ferroptosis sensitivity in ICC.

**Methods:**

Bioinformatic analyses of GEO and TCGA datasets identified ferroptosis‐related factors in ICC. MUC1 expression was validated in ICC cell lines and clinical specimens. Ferroptosis sensitivity was assessed through RSL3‐induced cell death assays, lipid peroxidation measurements, and iron detection. Mechanistic studies employed immunoprecipitation‐mass spectrometry, co‐immunoprecipitation, kinase assays, and deubiquitination assays. In vivo efficacy was evaluated using subcutaneous tumor models.

**Results:**

MUC1 was identified as a critical ferroptosis suppressor in ICC. MUC1 overexpression conferred RSL3 resistance by inhibiting lipid peroxidation and reducing ferrous iron accumulation, independent of the GPX4‐glutathione pathway. Mechanistically, MUC1 recruited Src kinase, which phosphorylated deubiquitinating enzyme ubiquitin‐specific protease 10 (USP10) at tyrosines 359 and 364, enhancing ferroptosis suppressor protein 1 (FSP1) deubiquitination at lysine 246 and stabilizing FSP1 protein. Concurrently, Src phosphorylated N‐myristoyltransferase 1 (NMT1) at tyrosine 41, augmenting FSP1 membrane localization through myristoylation. This dual mechanism potentiated the FSP1‐ coenzyme Q10 (CoQ10) antioxidant system. MUC1 knockdown significantly enhanced ferroptotic sensitivity in vitro and suppressed tumor growth in vivo.

**Conclusions:**

MUC1 orchestrates ferroptosis resistance in ICC through the Src‐USP10/NMT1‐FSP1 axis. Targeting this signaling cascade represents a potential therapeutic strategy for overcoming ferroptosis resistance in ICC.

**Key points:**

- MUC1 suppresses ferroptosis in ICC via Src‐mediated post‐translational modifications.
- Src phosphorylation of USP10 stabilizes FSP1 by removing K48‐linked polyubiquitin.
- Src activates NMT1 to enhance FSP1 myristoylation and membrane localization.

## Graphical abstract

MUC1 overexpression in intrahepatic cholangiocarcinoma (ICC) confers ferroptosis resistance by regulating FSP1. MUC1 activates Src kinase, which phosphorylates USP10, enhancing FSP1 stability via deubiquitination, and phosphorylates NMT1, promoting FSP1 membrane localisation through myristoylation. This dual mechanism potentiates the FSP1–CoQ10 axis, protecting against ferroptosis. Targeting the MUC1–Src–FSP1 pathway could offer novel therapeutic strategies for ICC.

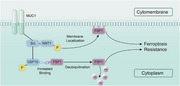

## 1 INTRODUCTION

Intrahepatic cholangiocarcinoma (ICC) is the second most common primary hepatic malignancy, accounting for approximately 10–15% of all primary liver cancers worldwide with steadily increasing incidence and mortality rates over recent decades.^1^, ^2^ Originating from the intrahepatic bile ducts, ICC is characterised by aggressive behaviour, late‐stage presentation and significant resistance to conventional therapeutic approaches. Despite advances in surgical techniques and systemic therapies, 5‐year survival rates remain dismal at 15–30%, emphasising the urgent need for novel therapeutic targets based on comprehensive understanding of ICC molecular pathophysiology.^3^, ^4^

Ferroptosis, a recently identified form of regulated cell death, is characterised by iron‐dependent accumulation of lipid reactive oxygen species (ROS) and subsequent cell membrane damage.^5^, ^6^, ^7^, ^8^ Unlike apoptosis or necrosis, ferroptosis is driven by the failure of cellular antioxidant systems, particularly glutathione peroxidase 4 (GPX4) and the cystine/glutamate antiporter system Xc^−^. Key regulatory mechanisms include iron metabolism dysregulation, lipid peroxidation of polyunsaturated fatty acids and depletion of reduced glutathione (GSH).^9^ Ferroptosis suppressor protein 1 (FSP1) functions as an alternative antioxidant system by reducing coenzyme Q10 (CoQ10) to protect cells from ferroptosis.^10^, ^11^ The unique metabolic dependencies of ferroptosis have positioned it as a promising therapeutic target in cancer treatment, particularly for malignancies resistant to conventional apoptosis‐inducing therapies.^12^, ^13^

Ferroptosis has been extensively investigated as a therapeutic strategy across multiple cancer types, providing valuable insights applicable to ICC research. In hepatocellular carcinoma, ferroptosis induction through GPX4 inhibition or system Xc^−^ disruption has demonstrated significant anti‐tumour efficacy in preclinical models.^14^ Sorafenib, a multi‐kinase inhibitor used in hepatocellular carcinoma treatment, was found to induce ferroptosis through inhibition of system Xc^−^, contributing to its therapeutic mechanism.^15^ Pancreatic adenocarcinoma studies have revealed that cancer cells develop ferroptosis resistance through up‐regulation of antioxidant systems, including enhanced CoQ10 biosynthesis and FSP1‐mediated lipid radical scavenging.^16^ In colorectal cancer, resistance mechanisms involve metabolic reprogramming of iron homeostasis and enhanced cysteine uptake.^17^ Translating these findings to cholangiocarcinoma reveals several parallels that support ferroptosis as a viable therapeutic target in ICC. Like other gastrointestinal malignancies, ICC exhibits altered iron metabolism, enhanced antioxidant capacity and dysregulated lipid metabolism – all processes central to ferroptosis regulation.^18^, ^19^ Post‐translational modifications (PTMs) play crucial roles in regulating protein function, localisation and stability across various cellular processes.^20^, ^21^ Recent studies have highlighted the importance of PTMs, particularly phosphorylation and ubiquitination, in modulating the ferroptosis pathway by affecting key regulators and determining cellular sensitivity to ferroptotic death.^22^, ^23^ In cancer cells, dysregulation of PTM‐related enzymes can contribute to ferroptosis evasion and therapeutic resistance.^24^ However, the specific PTM signalling networks regulating ferroptosis sensitivity in ICC remain poorly characterised.

Mucin 1 (MUC1) is a transmembrane glycoprotein normally expressed at the apical surface of epithelial cells that becomes overexpressed and aberrantly glycosylated in various epithelial tumours.^25^, ^26^ The MUC1 protein comprises a large extracellular N‐terminal domain and a smaller C‐terminal domain (MUC1‐C) functioning as a signalling molecule that interacts with various kinases and transcription factors to regulate downstream pathways involved in cell proliferation, survival and metastasis.^27^, ^28^, ^29^ In hepatobiliary malignancies, MUC1 overexpression is associated with tumour progression and poor prognosis.^30^ Despite its established role in multiple oncogenic processes, MUC1's potential involvement in regulating ferroptosis sensitivity in ICC has not been previously investigated.

We hypothesise that MUC1 plays a pivotal role in conferring ferroptosis resistance in ICC through regulation of PTM networks affecting key ferroptosis regulatory proteins. Mechanistically, we uncover a novel signalling axis in which MUC1 recruits and activates Src kinase, leading to phosphorylation of both ubiquitin‐specific protease 10 (USP10) and N‐myristoyltransferase 1 (NMT1). These phosphorylation events simultaneously stabilise FSP1 protein through deubiquitination and enhance its membrane localisation through myristoylation, thereby potentiating the FSP1–CoQ10 anti‐ferroptotic system. This study investigates the relationship between MUC1 expression and ferroptosis sensitivity in ICC cell lines and clinical specimens, elucidates the molecular mechanisms by which MUC1 regulates ferroptosis resistance through PTMs of FSP1 and evaluates the therapeutic potential of targeting the MUC1–FSP1 axis for overcoming ferroptosis resistance in ICC treatment. Understanding these mechanisms may reveal novel therapeutic vulnerabilities and provide a rationale for combination strategies targeting both MUC1 and ferroptosis pathways in ICC.

## 2 MATERIALS AND METHODS

### 2.1 Intracellular ferrous iron, lipid peroxidation and ROS detection

Intracellular ferrous iron (Fe^2+^) was detected using the FerroOrange probe (Dojindo, Kumamoto, Japan). Cells were treated as indicated, washed with phosphate buffered saline (PBS) and incubated with 1 µM FerroOrange for 30 min at 37°C. Fluorescence was observed under a fluorescence microscope (EVOS; Thermo Fisher Scientific, Waltham, USA).

Lipid peroxidation was assessed by measuring malondialdehyde (MDA) levels using a Lipid Peroxidation Assay Kit (Solarbio, Beijing, China) according to the manufacturer's instructions. Absorbance was measured at 532 nm using a microplate reader Infinite M200 (Tecan, Männedorf, Switzerland).

Intracellular ROS were detected using 2′,7′‐dichlorodihydrofluorescein diacetate (DCFH‐DA; Beyotime, Shanghai, China). Cells were incubated with 10 µM DCFH‐DA for 30 min at 37°C, washed with PBS and analysed by flow cytometry (Beckman Coulter, Brea, USA).

### 2.2 CoQ10 analysis

CoQ10 extraction and analysis were performed as previously described with minor modifications.^31^ Briefly, cells were harvested and homogenised in methanol:hexane (2:5, v/v). The mixture was centrifuged, and the hexane layer was collected and evaporated under nitrogen gas. The residue was resuspended in methanol and analysed by high‐performance liquid chromatography (HPLC) using a C18 column (Hepursen, Shenzhen, China) with electrochemical detection. The ratio of reduced CoQ10 to total CoQ10 was calculated based on peak areas.

### 2.3 Immunoprecipitation and mass spectrometry

For immunoprecipitation (IP), cells were lysed in IP buffer (20 mM Tris–HCl pH 7.4, 150 mM NaCl, 1% Triton X‐100 and protease/phosphatase inhibitors). Cell lysates (1 mg protein) were incubated with specific antibodies (2–5 µg) overnight at 4°C, followed by incubation with Protein A/G PLUS‐Agarose beads (Beyotime) for 2 h. The immunoprecipitates were washed five times with IP buffer, eluted with SDS sample buffer and subjected to SDS‐PAGE.

For IP–mass spectrometry (IP–MS), gel slices were excised and subjected to in‐gel trypsin digestion. Peptides were analysed using an Orbitrap Fusion Lumos mass spectrometer (Thermo Fisher Scientific) coupled to an EASY‐nLC 1200 system. MS data were processed using Proteome Discoverer software (version 2.4; Thermo Fisher Scientific) with the SEQUEST HT search engine against the UniProt human database.^32^

### 2.4 In vitro kinase assay

Recombinant active Src kinase (Sino Biological, Beijing, China) was incubated with purified USP10 or NMT1 proteins in kinase buffer (25 mM HEPES pH 7.5, 150 mM NaCl, 10 mM MgCl2, 2 mM DTT, 1 mM Na_3_VO_4_, 5 mM β‐glycerophosphate and 100 µM ATP) at 30°C for 30 min. Reactions were terminated by adding SDS sample buffer and analysed by Phos‐tag SDS‐PAGE or Western blot.

### 2.5 Deubiquitination assay

For cellular deubiquitination assays, HEK293 cells were co‐transfected with His‐FSP1, HA‐ubiquitin and Flag‐USP10 (wild‐type [WT] or mutants) for 48 h. Cells were treated with MG132 (10 µM) for 6 h before harvesting. Cell lysates were subjected to IP with anti‐His antibody, and ubiquitination levels were detected by Western blot using anti‐HA antibody.

For in vitro deubiquitination assays, Flag‐USP10 (WT or mutants) was immunopurified from transfected HEK293 cells. Enzymatic activity was measured using the ubiquitin‐7‐amino‐4‐methylcoumarin (Ub‐AMC) substrate (Abcam, Cambridge, UK).^33^ Reactions were performed in assay buffer (50 mM Tris–HCl pH 7.5, 150 mM NaCl, 2 mM DTT) at 37°C, and fluorescence (excitation/emission: 380/460 nm) was monitored using a microplate reader (Tecan).

### 2.6 Bioinformatic analysis

Transcriptomic data were obtained from the Gene Expression Omnibus (GEO) database (accession number GSE76297, containing 119 ICC samples and corresponding adjacent normal tissues) and The Cancer Genome Atlas (TCGA) cholangiocarcinoma dataset (36 tumour samples and nine normal samples). Raw data were normalised using the robust multi‐array average method. Differentially expressed genes (DEGs) between ICC and normal tissues were identified using the limma or DESeq2 package in R (version 4.1.0) with thresholds of |log2FC| > 1 and adjusted *p* value <.05. The Robust Rank Aggregation (RRA) algorithm was employed to integrate results from both datasets and identify consistently DEGs. Gene Set Enrichment Analysis (GSEA) was performed using the clusterProfiler package with ferroptosis‐related gene sets obtained from the Molecular Signatures Database (MSigDB, v7.4), including ‘KEGG_FERROPTOSIS’. A normalised enrichment score (NES) > 1.5 and false discovery rate < .25 were considered significant. Prediction of Src phosphorylation sites was conducted using NetPhos 3.1 software, and protein structure modelling was performed using AlphaFold‐Multimer. For prediction of ubiquitination sites in FSP1, we utilised MUSiteDeep (http://www.musite.net/). Prediction results were filtered using a stringent threshold score to identify high‐confidence ubiquitination sites.

### 2.7 Statistical analysis

Statistical analyses were performed using GraphPad Prism 10.0 software. Data are presented as mean ± standard deviation (SD) from at least three independent experiments. Differences between two groups were analysed using Student's *t*‐test, and multiple groups were compared using one‐way ANOVA followed by Tukey's post hoc test. *p* < .05 was considered statistically significant.

## 3 RESULTS

### 3.1 MUC1 overexpression in ICC functions as a ferroptosis regulatory factor

**FIGURE 1:**
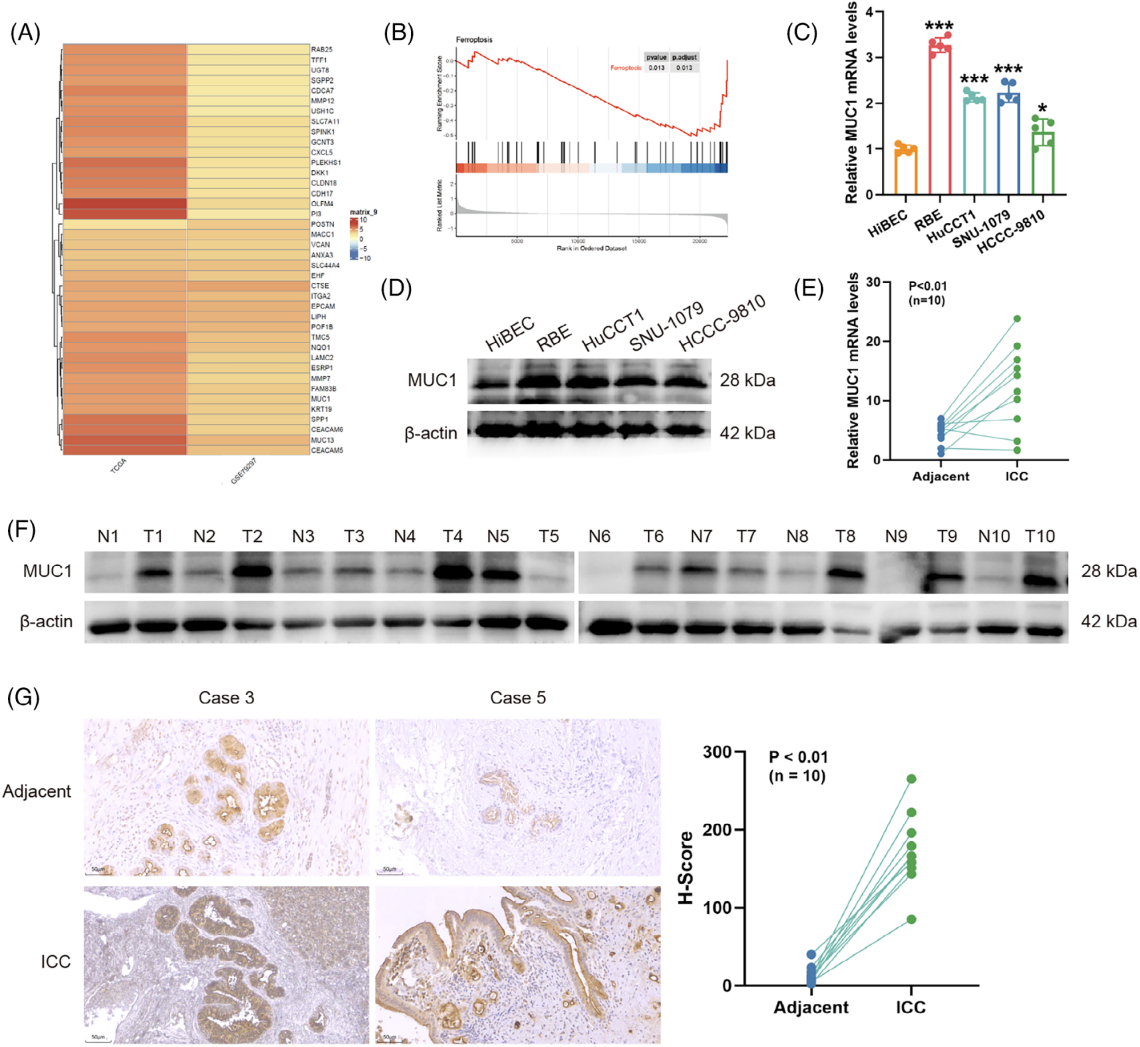
MUC1 is overexpressed in ICC and identified as a potential ferroptosis regulator. (A) Heatmap displaying differentially expressed genes between ICC and normal tissues identified through integration of GEO and TCGA datasets using Robust Rank Aggregation algorithm. (B) GSEA of ferroptosis‐related gene sets in MUC1‐high versus MUC1‐low ICC samples. (C) qRT‐PCR analysis of MUC1 mRNA expression in HiBEC and four ICC cell lines (RBE, HuCCT1, SNU‐1079, HCCC‐9810). (D) Western blot assessment of MUC1 protein expression in HiBEC and ICC cell lines. (E) qRT‐PCR analysis comparing MUC1 mRNA levels in 10 paired ICC tumour tissues and adjacent non‐tumour tissues. (F) Western blot analysis of MUC1 protein expression in representative paired clinical samples. (G) Representative immunohistochemical staining of MUC1 in ICC tumour tissues and adjacent non‐tumour tissues. Representative IHC images shown at 400× magnification; scale bar = 50 µm. Data are shown as the means ± SD, the significant level was identified by *p < .05; ***p < .001.

Integration and analysis of transcriptomic data from GEO and TCGA databases using RRA algorithm revealed significantly up‐regulated genes in intrahepatic ICC compared with normal tissues (Figure 1A). Intersection of the overexpressed DEGs with ferroptosis suppressor genes from FerrDb identified MUC1 as the sole uncharacterised molecule in ICC ferroptosis regulatory network (Figure S1A). GSEA demonstrated significant negative correlation between high MUC1 expression and ferroptosis‐related gene sets, indicating MUC1's inhibitory role in ferroptosis (Figure 1B).

Validation studies confirmed elevated MUC1 expression in ICC. Both mRNA and protein levels were significantly higher in four ICC cell lines (RBE, HuCCT1, SNU‐1079, HCCC‐9810) compared with normal biliary epithelial cells (HiBEC), as demonstrated by qRT‐PCR and Western blot (Figure 1C,D). Analysis of clinical samples from 10 ICC patients similarly showed increased MUC1 mRNA in tumour tissues versus adjacent non‐tumour tissues (Figure 1E), with further confirmation at protein level via Western blot (Figure 1F) and immunohistochemistry (Figure 1G). Correlation analyses demonstrated positive correlations between all three detection methods (qRT‐PCR vs. Western blot, qRT‐PCR vs. IHC *H*‐score and Western blot vs. IHC *H*‐score), confirming the consistency and reliability of MUC1 expression measurements across platforms (Figure S1B–D).

### 3.2 MUC1 confers protection against ferroptosis in ICC cells

**FIGURE 2:**
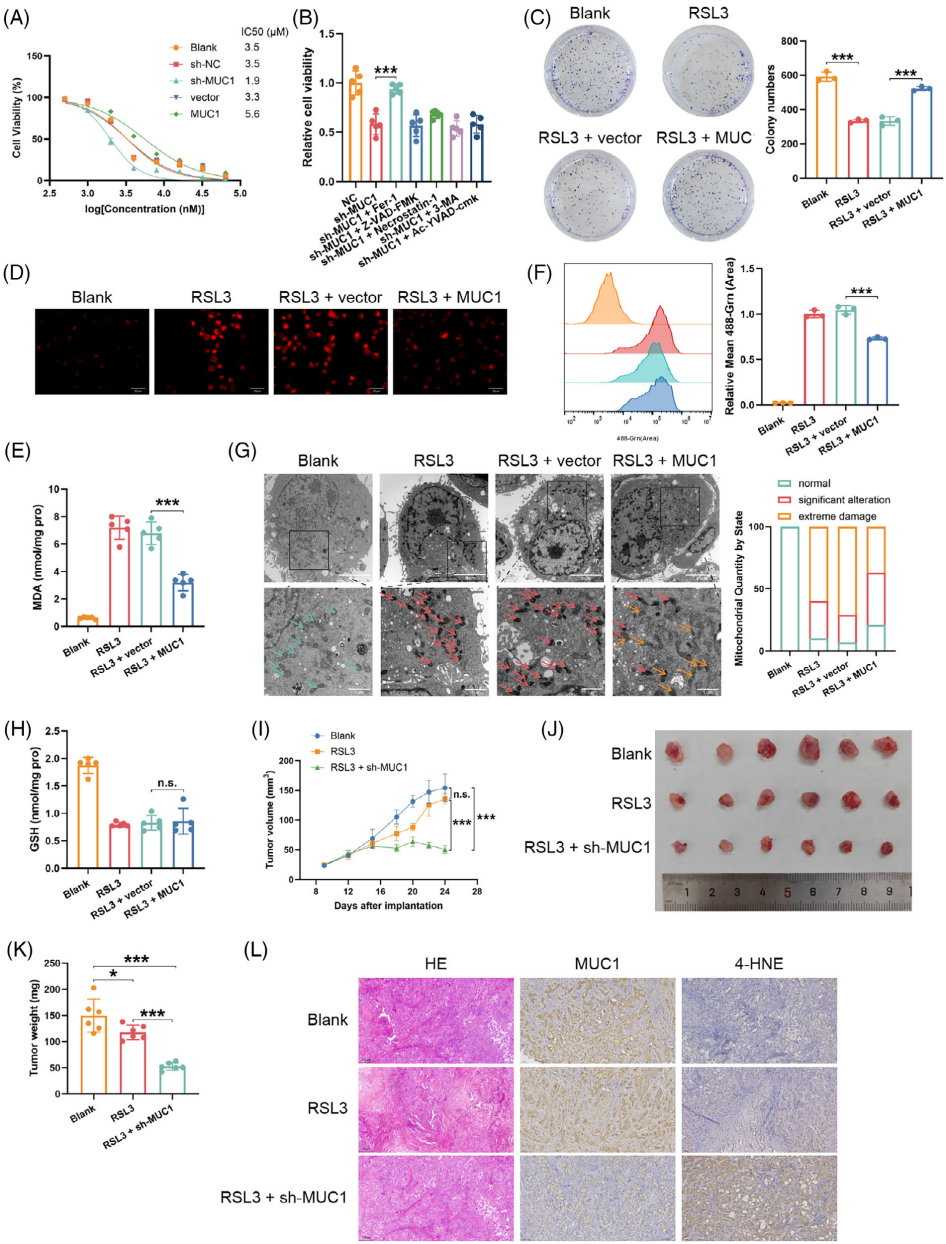
MUC1 confers resistance to ferroptosis in ICC cells through multiple mechanisms. (A) IC50 values of RSL3 (24 h treatment) in MUC1‐overexpressing and MUC1‐knockdown HuCCT1 cells compared with controls. Control experiments utilised sh‐NC (negative control short hairpin RNA), which does not target any known gene sequences and serves as an appropriate control for gene knockdown studies. (B) Cell viability of RSL3‐treated (2 µM, 24 h) sh‐MUC1 HuCCT1 cells following 1 h pre‐treatment with cell death inhibitors: ferroptosis inhibitor ferrostatin‐1 (5 µM), apoptosis inhibitor Z‐VAD‐FMK (30 µM), necroptosis inhibitor necrostatin‐1 (20 µM), autophagy inhibitor 3‐MA (5 mM) or pyroptosis inhibitor Ac‐YVAD‐cmk (20 µM). (C) Colony formation assay in HuCCT1 cells across treatment groups. (D) Ferrorrange staining for Fe^2^⁺ detection in HuCCT1 cells across treatment groups. Representative images shown at 200× magnification; scale bar = 25 µm. (E) Lipid peroxidation levels measured by MDA assay. (F) Intracellular ROS quantification by flow cytometry using DCFH‐DA staining. (G) Transmission electron micrographs showing mitochondrial morphology in HuCCT1 cells across treatment groups. Representative images at different magnifications: 1500× and 5000×; scale bars = 5 and 1 µm. (H) Intracellular GSH levels in HuCCT1 cells across treatment groups. (I–K) In vivo subcutaneous tumour model using HuCCT1 cells: (I) tumour growth curves, (J) representative tumour images and (K) endpoint tumour weights across treatment groups. (L) H&E, MUC1 and 4HNE immunohistochemical staining of tumour sections from different treatment groups. Representative images shown at 200× magnification; scale bar = 100 µm. Data are shown as the means ± SD, the significant level was identified by *p < .05; ***p < .001; n.s.: no significant.

Stable MUC1 overexpression and knockdown models were established in HuCCT1 and RBE cell lines to investigate MUC1's regulatory role in ICC ferroptosis, with validation at RNA and protein levels (Figure S1E–H). Drug sensitivity assays revealed MUC1 overexpression significantly increased IC50 values of RSL3, a specific GPX4 inhibitor that induces ferroptosis through covalent binding to the GPX4 active site, in both cell lines, while MUC1 knockdown reduced IC50 below control levels (Figures 2A and S2A), establishing a direct relationship between MUC1 and ferroptosis resistance. Among various cell death inhibitors, only the ferroptosis‐specific inhibitor Fer‐1 significantly reversed MUC1‐mediated protective effects (Figures 2B and S2B). Plate colony formation assays further demonstrated enhanced ICC cell survival under RSL3 stimulation with MUC1 overexpression (Figures 2C and S2C).

At the molecular level, MUC1 overexpression significantly reduced RSL3‐induced intracellular ferrous ion accumulation (Figures 2D and S2D), decreased cellular lipid peroxidation (Figures 2E and S2E) and reversed RSL3‐induced elevation of intracellular ROS (Figures 2F and S2F). Transmission electron microscopy revealed MUC1 overexpression attenuated RSL3‐induced mitochondrial alterations characteristic of ferroptosis (Figures 2G and S2G). However, MUC1 showed no significant effect on RSL3‐induced GSH depletion (Figures 2H and S2H), suggesting a non‐GPX4/GSH‐dependent mechanism.

In vivo validation using HuCCT1 cell‐derived subcutaneous tumour models demonstrated that MUC1 knockdown significantly reduced tumour volumes (Figure 2I–K), indicating enhanced tumour sensitivity to ferroptosis. Immunohistochemistry revealed increased 4HNE (a lipid peroxidation product and ferroptosis biomarker) staining in sh‐MUC1 group tumours, confirming elevated lipid peroxidation and enhanced ferroptosis (Figure 2L).

### 3.3 MUC1 inhibits ferroptosis through FSP1 stabilisation and membrane localisation

**FIGURE 3:**
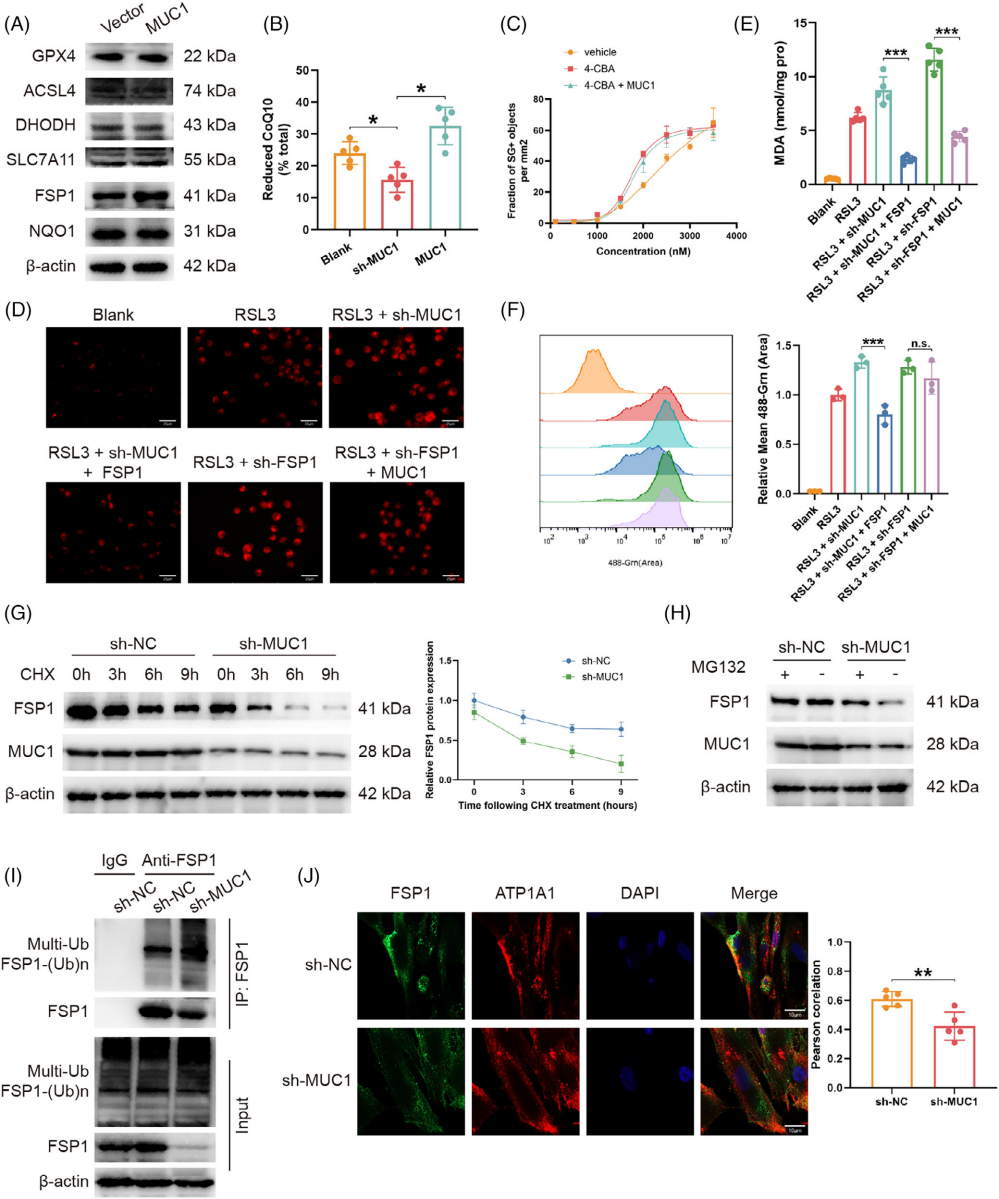
MUC1 regulates ferroptosis through FSP1 stabilisation and membrane localisation. (A) Western blot analysis of ferroptosis‐related proteins in HuCCT1 cells with or without MUC1 overexpression. (B) Ratio of reduced CoQ10 to total CoQ10 in HuCCT1 cells with MUC1 knockdown or overexpression, determined by HPLC. (C) SYTOX Green staining assessing cell death in HuCCT1 cells pretreated with 4‐CBA (3 mM, 24 h) followed by different RSL3 concentration exposure with or without MUC1 overexpression. (D–F) Ferroptotic phenotypes in HuCCT1 cells treated with RSL3 (2 µM, 24 h): (D) ferrous ion accumulation by Ferrorrange staining, representative images shown at 200× magnification; scale bar = 25 µm, (E) lipid peroxidation by MDA assay and (F) ROS levels by DCFH‐DA staining. (G) Western blot analysis of FSP1 protein degradation in HuCCT1 cells treated with cycloheximide (50 µg/mL) for indicated time periods following MUC1 knockdown. (H) Western blot analysis of FSP1 protein levels in HuCCT1 cells with MUC1 knockdown treated with or without MG132 (20 µM, 6 h). (I) Immunoprecipitation assay detecting FSP1 ubiquitination levels in HuCCT1 cells with MUC1 knockdown under MG132 (20 µM, 6 h) treatment. (J) Immunofluorescence co‐localisation analysis of FSP1 (green) and ATP1A1 (red) in HuCCT1 cells with MUC1 knockdown. Representative images shown at 600× magnification; scale bar = 10 µm. Data are shown as the means ± SD, the significant level was identified by *p < .05; **p < .01; ***p < .001; n.s.: no significant.

Western blot screening revealed that only FSP1 protein levels were significantly elevated in MUC1‐overexpressing cells, while other ferroptosis‐related proteins (GPX4, ACSL4, DHODH, SLC7A11) showed no significant changes (Figures 3A and S3A), identifying FSP1 as a critical downstream effector of MUC1‐regulated ferroptosis. Quantitative analysis of FSP1 expression was performed across multiple experimental platforms. Immunohistochemical evaluation of FSP1 in 10 paired ICC and adjacent normal tissue samples revealed significantly elevated *H*‐scores in ICC tissues (Figure S3B). Pearson correlation analysis demonstrated a significant positive correlation between MUC1 and FSP1 *H*‐scores in ICC tissues (Figure S3C), suggesting co‐regulation of these proteins in ICC pathogenesis. Cellular analysis confirmed FSP1 up‐regulation in ICC cell lines (Figure S3D). Western blot quantification of FSP1 protein levels in 10 paired ICC tissue samples demonstrated consistent elevation in ICC tissues relative to adjacent normal tissues (Figure S3E). As FSP1 inhibits ferroptosis by maintaining reduced CoQ10 levels, the impact of MUC1 on CoQ10 redox status was examined. MUC1 knockdown significantly decreased the proportion of reduced CoQ10, whereas MUC1 overexpression increased this ratio (Figures 3B and S3F). Intervention with CoQ10 synthesis inhibitor 4‐CBA demonstrated that MUC1 overexpression failed to rescue RSL3‐induced ferroptosis following 4‐CBA treatment (Figures 3C and S3G), establishing MUC1's protective effect dependence on the CoQ10 system.

Gene knockdown and compensation experiments established a causal relationship in the MUC1–FSP1 signalling pathway. Re‐expression of FSP1 in MUC1‐knockdown cells significantly reversed ferroptotic phenotypes, whereas MUC1 overexpression in FSP1‐knockdown cells failed to rescue ferroptosis‐related characteristics (Figures 3D–F and S3H–J). Subcutaneous tumour analysis further confirmed the functional relationship between MUC1 and FSP1‐mediated ferroptosis resistance. Tumours derived from sh‐MUC1 cells exhibited reduced ratios of reduced CoQ10, indicating compromised antioxidant capacity (Figure S3K). Western blot analysis of subcutaneous tumour tissues confirmed decreased FSP1 protein levels in sh‐MUC1 tumours compared with control tumours (Figure S3L).

MUC1 regulation of FSP1 occurred primarily at the protein level, as MUC1 knockdown or overexpression had no significant effect on FSP1 mRNA expression (Figure S4A). Cycloheximide treatment demonstrated MUC1 knockdown significantly accelerated FSP1 protein degradation (Figures 3G and S4B). Proteasome inhibitor MG132 treatment prevented FSP1 decrease under MUC1 knockdown conditions (Figures 3H and S4C), while autophagy inhibitor BafA1 showed no significant effect (Figure S4D), indicating ubiquitin‐proteasome pathway‐mediated degradation. Co‐IP confirmed increased FSP1 ubiquitination levels with MUC1 knockdown under MG132 treatment (Figures 3I and S4E).

Immunofluorescence and cellular fractionation analyses revealed MUC1 knockdown significantly reduced FSP1‐membrane co‐localisation (Figures 3J and S4F,G), demonstrating MUC1 both stabilises FSP1 protein and promotes its membrane localisation to enhance anti‐ferroptotic activity.

### 3.4 MUC1 regulates FSP1 stability through Src‐mediated USP10 phosphorylation

**FIGURE 4:**
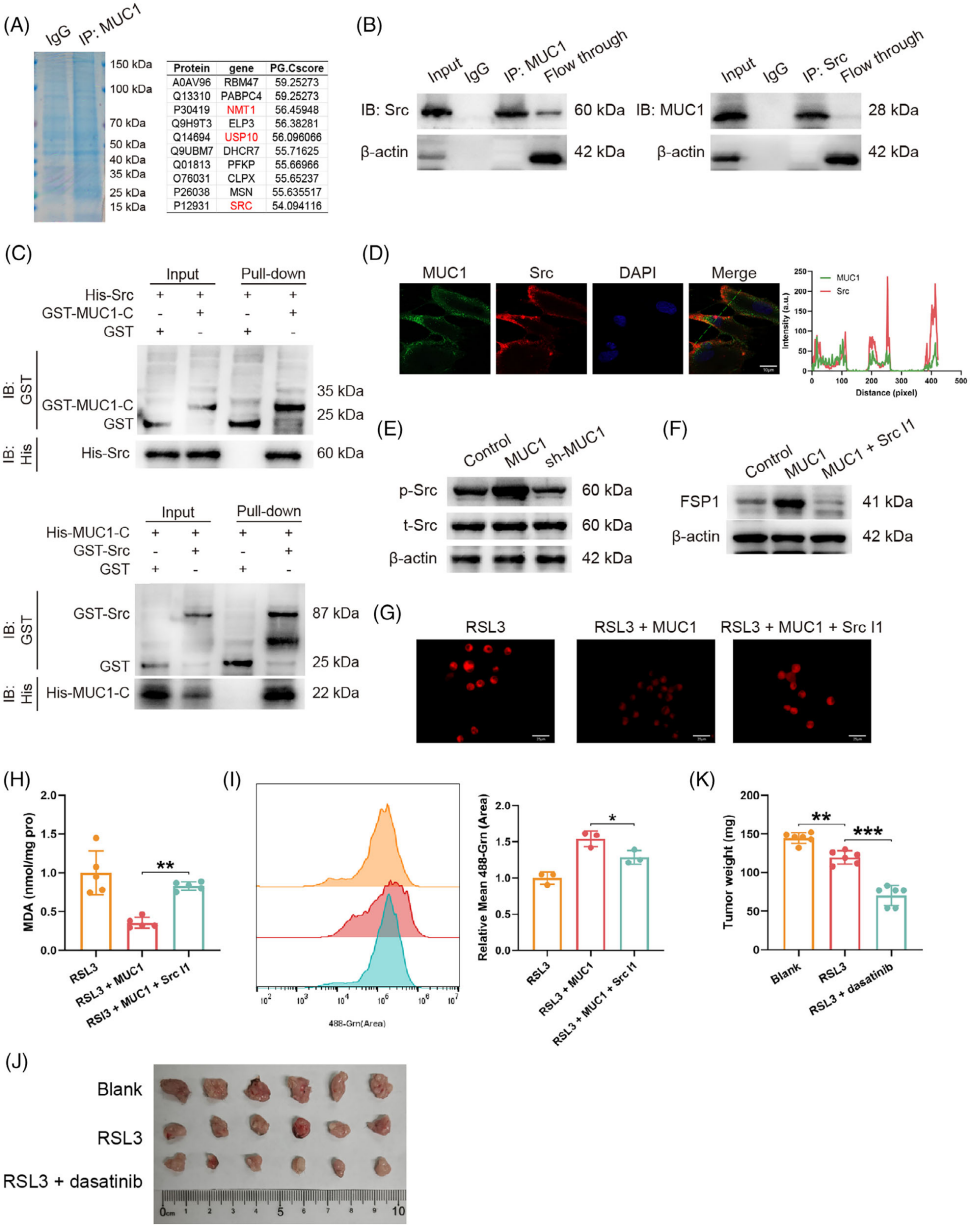
MUC1 interacts with and activates Src kinase to regulate FSP1 expression. (A) MUC1 interactome identification in HuCCT1 cells: left panel shows Coomassie blue staining of immunoprecipitated proteins; right panel displays tabulated IP–MS results of MUC1‐associated proteins. (B) Co‐immunoprecipitation assays demonstrating interaction between MUC1 and Src in HuCCT1 cells. (C) In vitro GST pull‐down assay confirming direct binding between recombinant MUC1 and Src proteins. (D) Immunofluorescence co‐localisation analysis of MUC1 (green) and Src (red) in HuCCT1 cells. Representative images shown at 600× magnification; scale bar = 10 µm. (E) Western blot analysis of Src phosphorylation at Tyr419 in HuCCT1 cells with MUC1 manipulation. (F) Western blot analysis of FSP1 protein levels in HuCCT1 cells with MUC1 overexpression treated with or without Src inhibitors. (G) Ferrorrange staining for ferrous ion detection in HuCCT1 cells under indicated treatments. Representative images shown at 200× magnification; scale bar = 25 µm. (H) Lipid peroxidation levels measured by MDA assay. (I) Flow cytometric analysis of intracellular ROS levels using DCFH‐DA staining. (J) Representative images of subcutaneous tumours from different treatment groups: control, RSL3 treatment and RSL3 + dasatinib combination therapy. (K) Quantitative analysis of tumour weights across treatment groups. Data are shown as the means ± SD, the significant level was identified by *p < .05; **p < .01; ***p < .001.

IP–MS analysis identified Src kinase and deubiquitinating enzyme USP10 as MUC1‐interacting proteins (Figure 4A and Table S1). Co‐IP and GST pull‐down assays confirmed direct binding between MUC1 and Src (Figures 4B,C and S5A), with immunofluorescence showing predominant co‐localisation at the cell membrane (Figures 4D and S5B). MUC1 overexpression significantly increased phosphorylation at Src active site Tyr419 without affecting total Src levels, while knockdown reduced this phosphorylation (Figures 4E and S5C), demonstrating MUC1 promotes Src activation, potentially through enhancement of its autophosphorylation. To confirm the role of Src in the MUC1–FSP1 axis, Western blot analysis showed that MUC1 overexpression significantly increased FSP1 protein levels, but this effect was abrogated by Src inhibition, indicating that Src activity is essential for MUC1‐mediated FSP1 stabilisation (Figures 4F and S5D). To determine whether Src inhibition reverses the anti‐ferroptotic effects of MUC1, various ferroptotic markers were examined. MUC1 overexpression was observed to significantly reduce RSL3‐induced Fe^2^⁺ accumulation, lipid peroxidation and ROS generation. However, treatment with Src inhibitor I1 abolished these protective effects (Figure 4G–I). In vivo therapeutic efficacy was evaluated using subcutaneous tumour models. Tumour morphology demonstrated visible reduction in tumour size with combination treatment (Figure 4J). Quantitative assessment revealed significantly reduced tumour weights in the RSL3 and dasatinib combination group compared with RSL3 monotherapy and control groups (Figure 4K).

**FIGURE 5:**
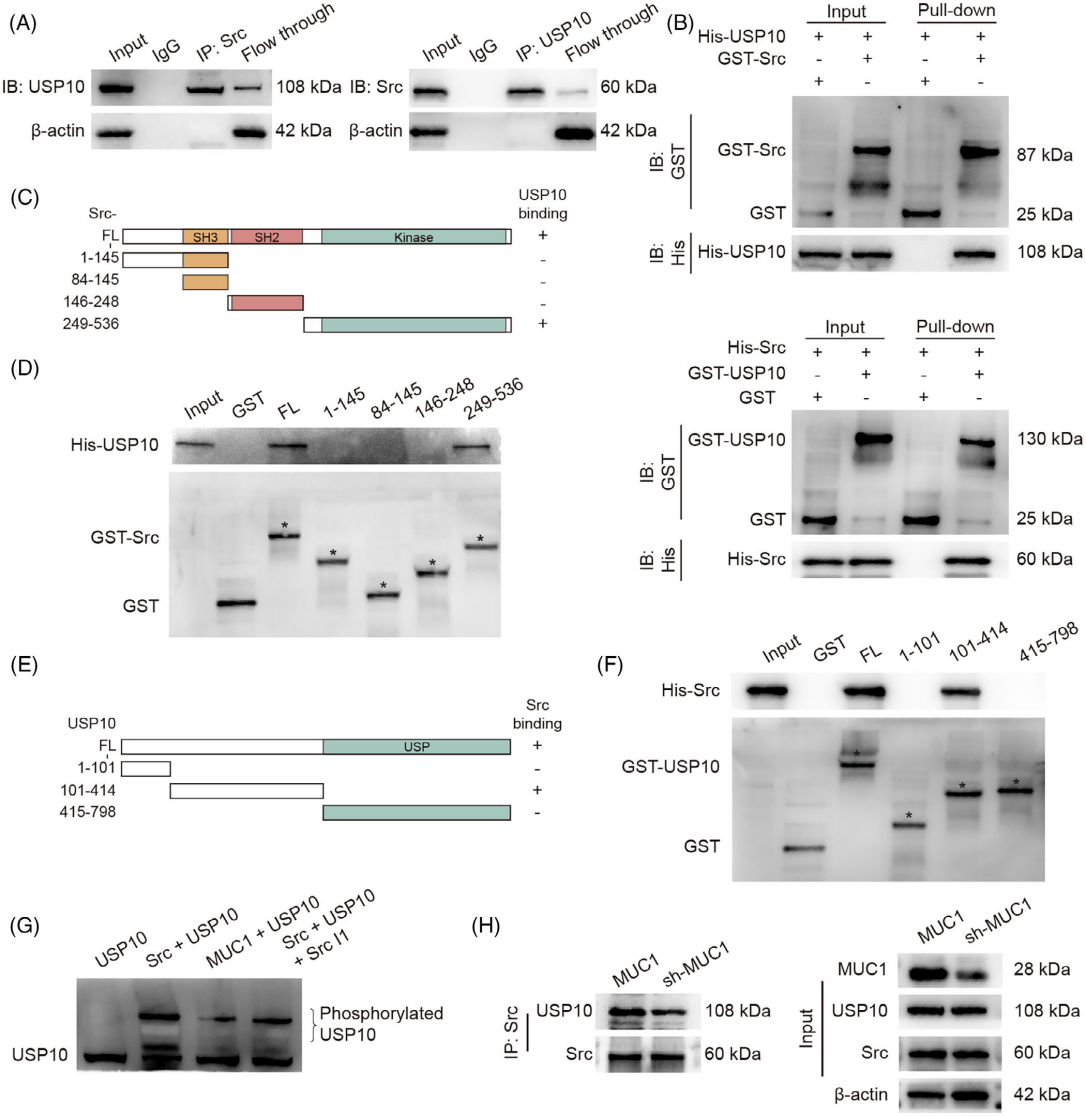
Src phosphorylates USP10 to regulate FSP1 stability. (A) Co‐immunoprecipitation assays demonstrating interaction between Src and USP10 in HuCCT1 cells. (B) In vitro GST pull‐down assay confirming direct binding between recombinant Src and USP10 proteins. (C–F) Domain mapping experiments identifying critical interaction regions: (C) schematic of Src domain mutants, (D) GST pull‐down assay with Src mutants, (E) schematic of USP10 domain mutants, (F) GST pull‐down assay with USP10 mutants. (G) Phos‐tag SDS‐PAGE analysis of USP10 phosphorylation status following in vitro kinase reactions with purified Src, MUC1 and Src inhibitor. (H) Co‐immunoprecipitation assays examining Src–USP10 interaction in HuCCT1 cells with MUC1 knockdown.

MS of FSP1 immunoprecipitated identified USP10, corroborating the findings from MUC1 IP–MS results (Figure S5E and Table S2), with Co‐IP and GST pull‐down confirming Src–USP10 interaction (Figures 5A,B and S5F). Domain deletion analysis identified Src kinase domain (249–536) and USP10 amino acid sequence 101–414 as essential interaction regions (Figure 5C–F). In vitro experiments, Phos‐tag™ SDS‐PAGE revealed Src effectively phosphorylated USP10, with this effect blocked by Src inhibitor (Figure 5G). Src overexpression resulted in increased USP10 phosphorylation, whereas treatment with Src inhibitors blocked this effect. Conversely, MUC1 knockdown reduced USP10 phosphorylation, and subsequent Src overexpression did not significantly restore phosphorylation levels. These findings demonstrate that MUC1 is required for Src‐mediated USP10 phosphorylation (Figure S5G,H). MUC1 knockdown significantly reduced Src–USP10 binding (Figures 5H and S5I), indicating that MUC1 functions as a scaffold protein facilitating Src–USP10 interaction, thereby regulating USP10 phosphorylation and FSP1 stability.

### 3.5 Src‐mediated USP10 phosphorylation enhances FSP1 deubiquitination

**FIGURE 6:**
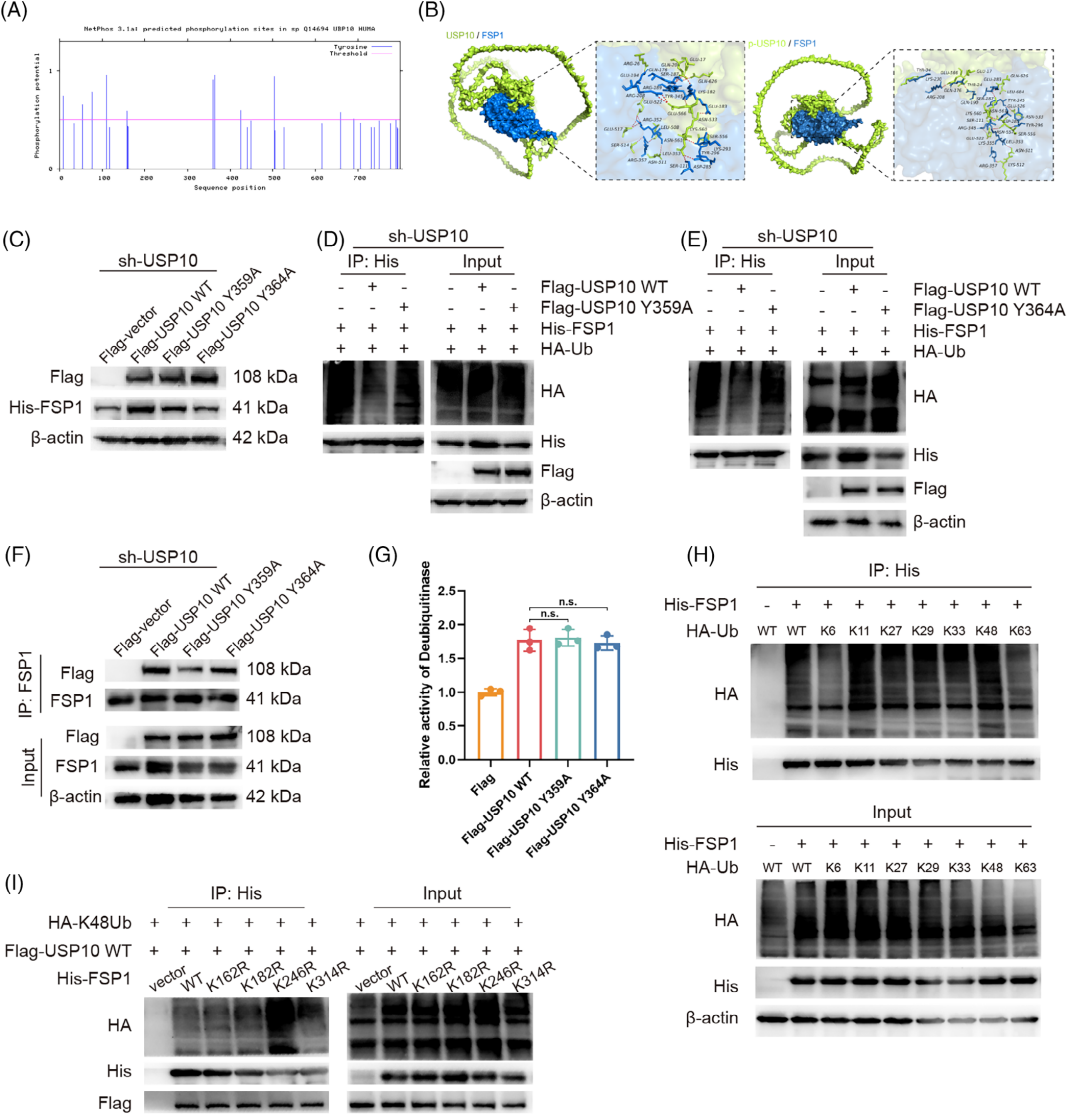
Src‐mediated USP10 phosphorylation and its effect on FSP1 deubiquitination. (A) Prediction of potential Src phosphorylation sites on USP10 using Netphos 3.1. (B) Molecular docking analysis of USP10 (wild‐type and phosphorylated Y359/Y364) with FSP1 protein using AlphaFold‐Multimer. (C) Western blot analysis examining His‐tagged FSP1 levels in USP10‐knockdown HEK293 cells expressing wild‐type or mutant (Y359A/Y364A) Flag‐USP10. (D and E) Assessment of FSP1 polyubiquitination in cells expressing wild‐type or mutant USP10 following MG132 treatment (10 µM, 6 h). (F) Co‐immunoprecipitation analysis of FSP1 with wild‐type or mutant USP10. (G) In vitro enzymatic activity measurement of purified wild‐type and mutant USP10 using Ub‐AMC substrate. (H) Analysis of ubiquitin chain types on FSP1 using ubiquitin vectors containing single lysine mutations. (I) Identification of FSP1 ubiquitination sites through point mutation analysis. Data are shown as the means ± SD, with statistical significance indicated as n.s.: not significant.

To elucidate the precise regulatory mechanism of USP10 in the ferroptosis regulatory network, Netphos 3.1 was employed to predict potential Src phosphorylation sites within USP10. Analysis revealed high‐confidence sites (scoring above .9) including 110Y, 359Y, 364Y and 503Y (Figure 6A). Phosphorylation levels were subsequently validated through Phos‐tag gel electrophoresis, which demonstrated that mutation of tyrosine residues at positions 359 and 364 to alanine (Y359A or Y364A) significantly reduced USP10 phosphorylation under Src kinase activity. The simultaneous mutation of both sites (Y359A/Y364A) exhibited a more pronounced inhibitory effect on phosphorylation (Figure S6A), thereby confirming Y359 and Y364 as critical Src phosphorylation sites on the USP10 protein.

AlphaFold‐Multimer predictions revealed phosphorylated USP10 (pY359/pY364) exhibited increased interaction probability with FSP1 compared with WT (ipTM scores: .62 vs. .43) (Figures 6B and S6B). To validate this prediction, USP10 expression plasmids with Y359A/Y364A point mutations were constructed. In USP10‐knockdown HEK293 cells, overexpression of Flag‐tagged WT USP10 stabilised His‐tagged FSP1 protein levels, whereas USP10 mutants Y359A or Y364A failed to retain this function (Figure 6C). Further experiments demonstrated that Flag‐USP10 WT stabilised FSP1 protein by removing polyubiquitination modifications, while Flag‐USP10 Y359A or Y364A mutants exhibited diminished deubiquitinating activity (Figure 6D,E).

Co‐IP confirmed reduced USP10–FSP1 interaction with USP10 (Y359A) and USP10 (Y364A) mutants (Figure 6F), without altering USP10's deubiquitinase activity (Figure 6G), indicating phosphorylation primarily affects USP10's binding affinity to FSP1. Further analysis determined FSP1 is predominantly modified through K11 and K48 ubiquitin chain linkages (Figure 6H), with K246 identified as the critical site for K48 polyubiquitination and primary target for USP10's deubiquitinating function (Figures 6I and S6C). Additional structural analyses revealed direct interaction between USP10's USP domain (415–798) and FSP1's C‐terminal domain (318–373) (Figure S6F–I).

### 3.6 Src‐mediated NMT1 phosphorylation regulates FSP1 membrane localisation

**FIGURE 7:**
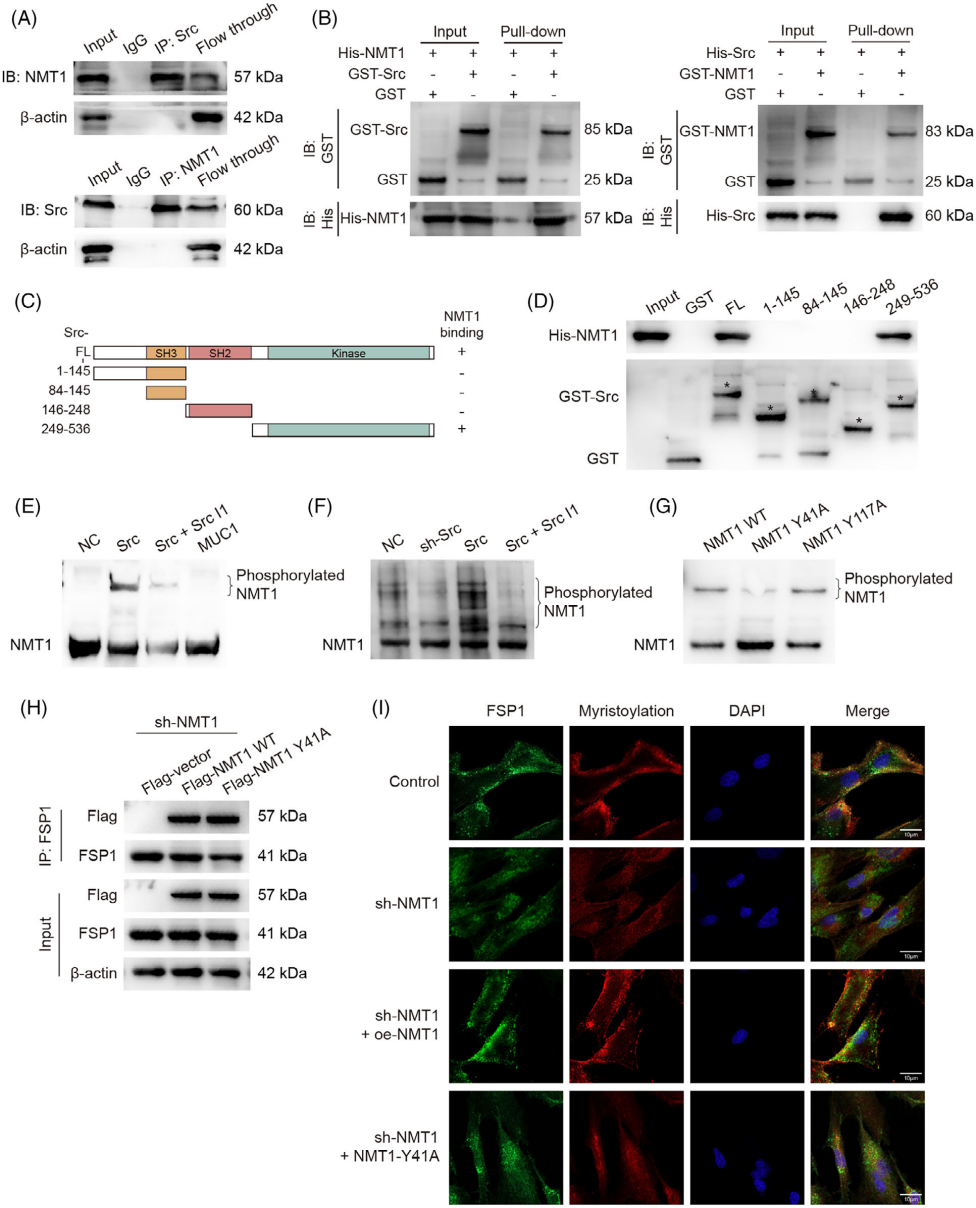
Src and NMT1 interaction and their effects on FSP1 localisation. (A) Co‐immunoprecipitation analysis of Src and NMT1 in HuCCT1 cells. (B) GST pull‐down assay with purified Src and NMT1 proteins. (C) Schematic illustration of Src domain constructs used for interaction mapping. (D) GST pull‐down assay using different Src domain constructs with NMT1. (E) Phos‐tag gel electrophoresis of NMT1 following in vitro kinase reactions under various conditions including Src, Src inhibitor and MUC1. (F) Phos‐tag analysis of NMT1 in cells with Src manipulation and inhibitor treatment. (G) Phos‐tag analysis comparing wild‐type and Y41A mutant NMT1 after Src kinase reaction. (H) Co‐immunoprecipitation analysis between FSP1 and either wild‐type or Y41A mutant NMT1. (I) Fluorescence microscopy visualisation of cellular myristoylation (red) and FSP1 distribution (green) in cells expressing wild‐type or Y41A mutant NMT1. Representative images shown at 600× magnification; scale bar = 10 µm.

NMT1 knockdown in HuCCT1 cells significantly enhanced RSL3 sensitivity, reducing IC50 values compared with negative control (Figure S7A). NMT1 inhibition promoted ferroptosis markers, with elevated MDA levels and increased Fe^2+^ accumulation in RSL3‐treated cells (Figure S7B,C), confirming enhanced iron‐dependent oxidative stress upon NMT1 depletion. Co‐IP confirmed stable Src–NMT1 interaction in ICC cells (Figures 7A and S7D). GST pull‐down experiments demonstrated direct Src–NMT1 binding, with Src kinase domain identified as the critical interaction region (Figure 7B–D). Phos‐tag gel electrophoresis confirmed Src significantly increased NMT1 phosphorylation, an effect attenuated by Src inhibition, while MUC1 showed no direct effect on NMT1 phosphorylation (Figure 7E). Cellular validation demonstrated positive correlation between NMT1 phosphorylation and Src expression levels, with phosphorylation effectively inhibited by Src inhibitors (Figures 7F and S7E).

MS identified tyrosine (Y) 41 and 117 as likely Src phosphorylation targets on NMT1, with Phos‐tag validation confirming Y41 as a critical phosphorylation site (Figure 7G). Y41A mutation did not affect NMT1–FSP1 binding (Figure 7H), suggesting phosphorylation regulates function through NMT1's catalytic activity rather than protein interaction.

Fluorescence microscopy demonstrated NMT1 inhibition significantly reduced cellular myristoylation levels. While WT NMT1 overexpression restored myristoylation, Y41A‐mutated NMT1 failed to effectively restore these modifications. FSP1 membrane localisation increased with elevated myristoylation levels (Figures 7I and S7F), with cellular fractionation confirming increased membrane‐associated FSP1 proportion with higher myristoylation levels (Figures S7G and 8).

**FIGURE 8:**
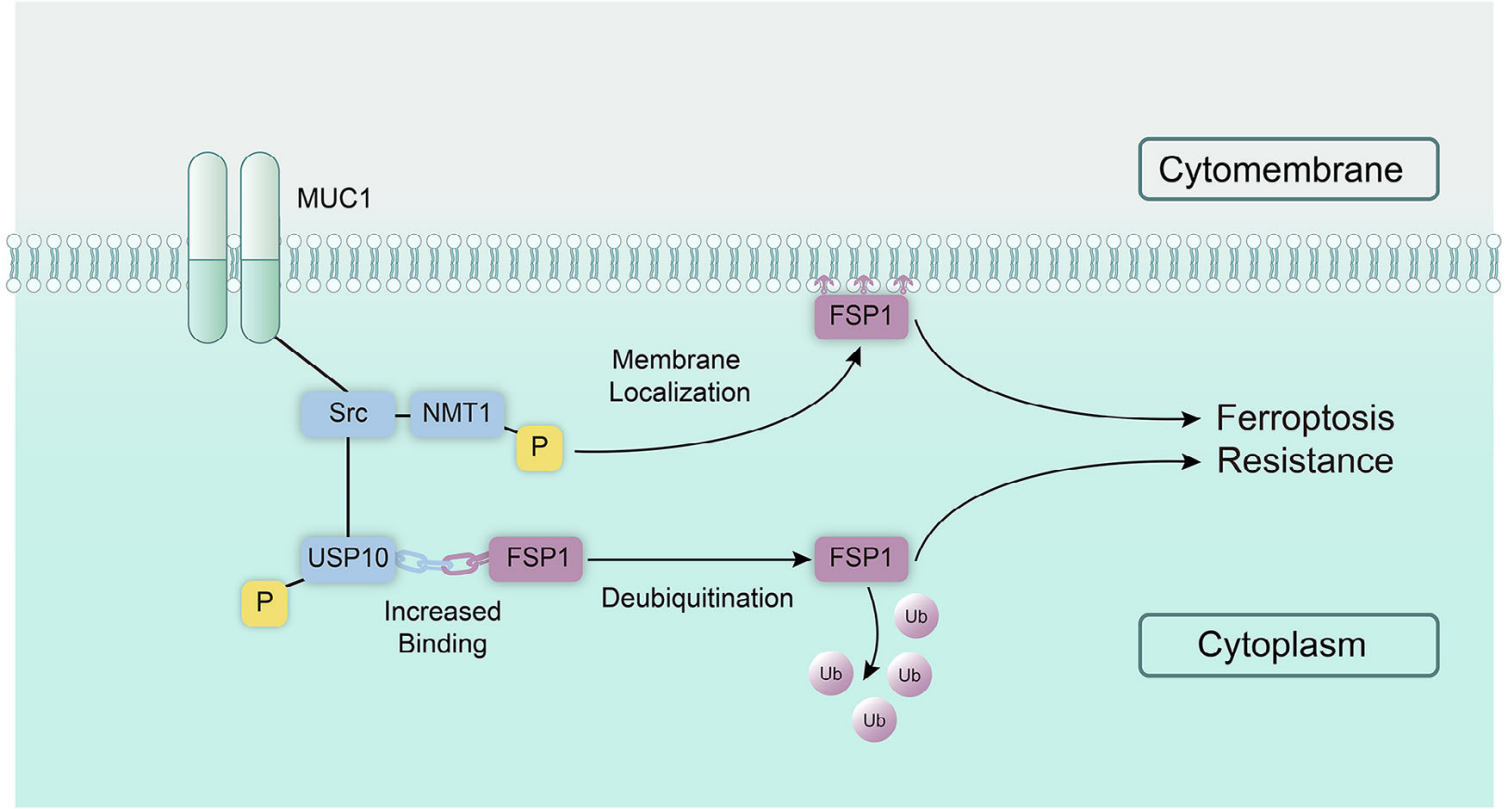
MUC1 promotes ferroptosis resistance in ICC cells through dual regulatory mechanisms targeting FSP1. MUC1 overexpression in ICC cells results in Src kinase activation, leading to the phosphorylation of two critical regulatory proteins: (1) USP10 at tyrosine residues Y359/Y364, which enhances its deubiquitinase activity and strengthens its binding affinity toward FSP1, thereby stabilising FSP1 protein levels in the cytoplasm; and (2) NMT1 at tyrosine residue Y41, which increases its myristoyltransferase activity and facilitates N‐terminal myristoylation and subsequent membrane localisation of FSP1. Membrane‐localised FSP1 efficiently catalyses CoQ10 reduction, establishing a lipid peroxide‐trapping system that neutralises lipid peroxidation and prevents ferroptotic cell death. Through these dual regulatory mechanisms (protein stabilisation and subcellular localisation), the FSP1–CoQ10 antioxidant axis is potentiated by MUC1, conferring ferroptosis resistance to ICC cells.

## 4 DISCUSSION

This study identifies MUC1 as a critical regulator of ferroptosis resistance in ICC, establishing a novel PTM signalling network that controls ferroptotic cell death. Our findings reveal that MUC1 orchestrates ferroptosis inhibition through dual mechanisms: stabilising FSP1 protein via Src–USP10‐mediated deubiquitination and enhancing FSP1 membrane localisation through Src–NMT1‐dependent myristoylation.

Ferroptosis has emerged as a promising therapeutic target in multiple cancer types, including ICC.^34^, ^35^, ^36^ While previous studies have focused on transcriptional regulation of ferroptosis‐related genes, the present work highlights the critical importance of PTMs in modulating ferroptotic sensitivity.^37^, ^38^, ^39^ The identification of MUC1 as a ferroptosis suppressor in ICC represents a significant advance in understanding the molecular mechanisms underlying treatment resistance in this aggressive malignancy.

MUC1 was selected as our primary research target based on its well‐documented overexpression in ICC and established oncogenic functions across multiple cancer types. The molecular weight of the detected MUC1 fragment corresponds to the MUC1‐C, which is generated following proteolytic cleavage of the full‐length MUC1 protein at the cell surface. This fragment, approximately 70 amino acids in length and lacking the glycosylated extracellular domain, plays a crucial role in intracellular signalling. In cancer cells, MUC1‐C regulates pathways such as ferroptosis, contributing to cell survival and chemoresistance.

FSP1 demonstrated elevated protein expression in both ICC cell lines and tissue samples, showing significant positive correlation with MUC1 expression levels. Through systematic screening of ferroptosis regulatory factors, FSP1 was identified as the primary mediator of MUC1's anti‐ferroptotic effects.^40^, ^41^ This conclusion was supported by unchanged GSH levels and altered CoQ10 redox status, indicating that MUC1 functions through the FSP1–CoQ10 axis rather than the canonical GPX4 pathway.

The MUC1–Src interaction represents a critical upstream regulatory node in this pathway. Src kinase activation by MUC1 initiates downstream signalling cascades that ultimately converge on FSP1 regulation, demonstrating the hierarchical organisation of this ferroptosis resistance mechanism. The Src–USP10–FSP1 axis represents a previously unrecognised regulatory pathway in ferroptosis. Y359 and Y364 were identified as critical Src phosphorylation sites on USP10 that enhance its binding affinity for FSP1, facilitating the removal of K48‐linked polyubiquitin chains at the K246 site of FSP1. This deubiquitination event stabilises FSP1 protein levels, thereby maintaining the CoQ10 redox system that neutralises lipid peroxides.

The MUC1–Src–NMT1 axis represents an equally important regulatory mechanism. Src phosphorylation of NMT1 at Y41 enhances its NMT activity, facilitating its membrane localisation and subsequent anti‐ferroptotic function. Our investigation of NMT1 was guided by its identification as a MUC1‐interacting protein in our IP–MS analysis and its established role in protein myristoylation.^42^ Previous studies by Bersuker et al.^31^ demonstrated that N‐terminal myristoylation by NMT1 is essential for FSP1's membrane localisation and subsequent anti‐ferroptotic function through the CoQ10 redox system. This critical PTM provides a mechanistic link between MUC1, FSP1 trafficking and ferroptosis resistance, further supporting our model of MUC1 as a multi‐faceted regulator of FSP1 activity through both protein stabilisation and proper subcellular localisation. Additionally, sh‐NMT1 treatment altered ICC cell IC50 values and affected MDA and Fe^2+^ levels, promoting ferroptosis and confirming NMT1's critical role in ferroptosis resistance.

The clinical relevance of these findings is supported by the observed overexpression of MUC1 in ICC patient samples and its correlation with ferroptosis resistance. This suggests that MUC1 expression levels could serve as a biomarker for predicting ferroptosis sensitivity in ICC patients. Moreover, the in vivo experiments demonstrating enhanced ferroptotic sensitivity in MUC1‐knockdown tumours provide proof‐of‐concept for targeting MUC1 or its downstream effectors to overcome ferroptosis resistance in ICC. It is worth noting that in the subcutaneous tumour experiments, HuCCT1 cells were utilised due to the failure of RBE to form tumours, highlighting the necessity for alternative models in this investigation.

Several limitations of this study warrant consideration. While MUC1 has been established as a ferroptosis regulator in ICC, its role in other cancer types remains to be investigated. RBE and HuCCT1 cell lines were selected based on their high MUC1 expression, widespread use in ICC research and presence of KRAS mutations associated with ferroptosis mechanisms in ICC. While these models represent a common subset of ICC, results may vary in other ICC subtypes due to different molecular characteristics. Further studies in additional ICC cell lines may help establish broader applicability of our findings. Additionally, the potential crosstalk between the MUC1–FSP1 axis and other ferroptosis regulatory pathways, such as the GPX4–GSH system, requires further exploration. Future studies should also address the potential therapeutic implications of targeting this pathway in patient‐derived xenograft models and clinical trials.

Future investigations should focus on developing therapeutic strategies targeting the MUC1–FSP1 axis. Given the overexpression of MUC1 in ICC, targeting MUC1 with antibody–drug conjugates (ADCs) or other therapeutic strategies holds great promise. ADCs targeting MUC1 are currently being tested in clinical trials for various cancers, and similar approaches could be explored in ICC. Additionally, combination therapies incorporating ferroptosis inducers with MUC1 or FSP1 inhibitors may provide synergistic therapeutic benefits for ICC patients. Beyond ICC, the MUC1–Src–FSP1 axis may have broader therapeutic implications for other MUC1‐overexpressing malignancies. In breast cancer (∼90% MUC1‐positive) and pancreatic ductal adenocarcinoma (>80% MUC1‐positive), similar ferroptosis resistance mechanisms may operate through this pathway. The conserved nature of Src signalling and ubiquitous expression of USP10 and NMT1 suggest this regulatory network could be functionally relevant across cancer types, potentially representing a broadly applicable strategy for overcoming therapeutic resistance in MUC1‐positive cancers.

## AUTHOR CONTRIBUTIONS

Z. Y. Q. and Z. X. Y. conceived the study. Z. Y. Q., Y. S. F., H. L., L. X. Y. and H. Q. X. performed the experiments and data analyses. N. Q. C., L. S. Y., Z. C. L., S. B. S. and Y. Y. M. analysed and interpreted the data. Z. Y. Q. and S. B. S. wrote the manuscript.

## CONFLICT OF INTEREST STATEMENT

The authors declare no conflicts of interest.

## ETHICS STATEMENT

All participants provided informed consent. All human tissue research in this study had the approval of ethics committees of the Second Affiliated Hospital of Harbin Medical University (Harbin, China). All animal experiments were performed in accordance with a protocol approved by the Institutional Animal Care and Use Committee of the Second Affiliated Hospital of Harbin Medical University.

## Supporting information



Supporting Information

## Data Availability

All data generated or analysed during this study are included in the article and its Supplementary Information files or are available from the corresponding author upon reasonable request.
