# Supplementary material for: MUC1 drives ferroptosis resistance in ICC via Src‐mediated FSP1 deubiquitination and myristoylation
Source: Clin Transl Med. 2025 Oct 9;15(10):e70495. doi: 10.1002/ctm2.70495 (PMC12510807; doi:10.1002/ctm2.70495)
Supplement: Supplementary file 1 — Supporting Information [file CTM2-15-e70495-s001.docx]

**Supplemental information**

**Supplementary Figures**


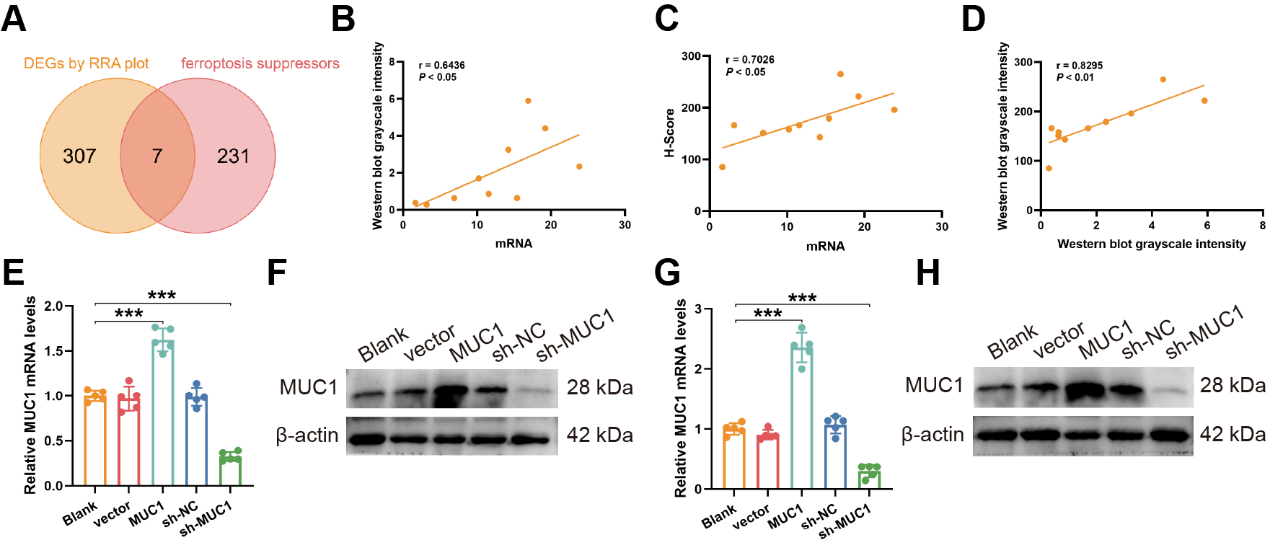


**Figure S1** Identification of MUC1 as a ferroptosis regulator and validation of genetic manipulation models. (A) Venn diagram showing intersection of overexpressed DEGs in ICC with ferroptosis suppressor genes from FerrDb database. (B-D) Pearson correlation analysis between (B) MUC1 qRT-PCR and Western blot quantification results, (C) MUC1 qRT-PCR and immunohistochemical H-Score quantification and (D) MUC1 Western blot quantification and IHC H-Score in ICC tissue samples (n=10 pairs). (E-H) Validation of MUC1 overexpression and knockdown in HuCCT1 (E, F) and RBE (G, H) cell lines at mRNA and protein levels by qRT-PCR (E, G) and Western blot (F, H). Data are shown as the means ± SD, the significant level was identified by *** *P* < 0.001.


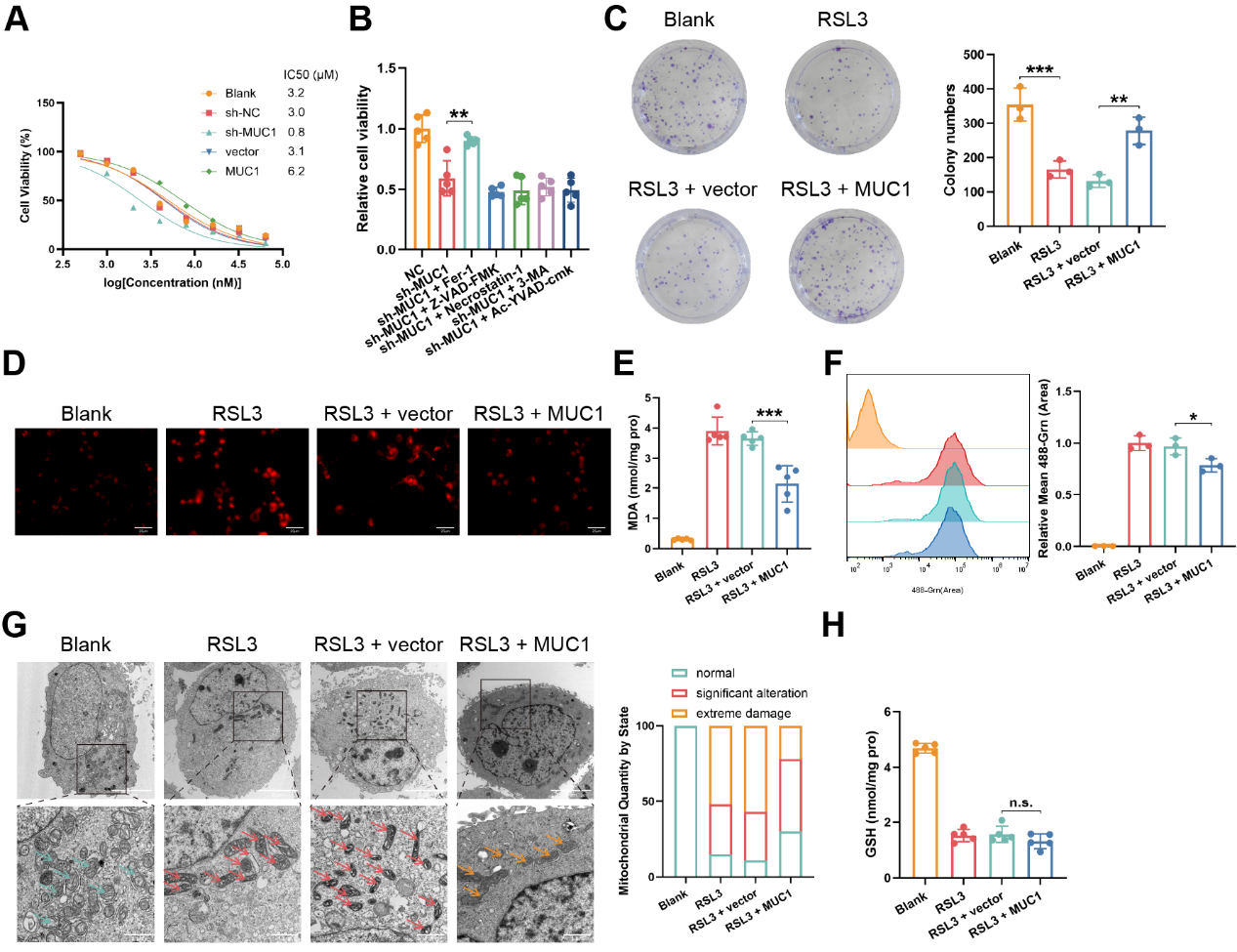


**Figure S2** MUC1 inhibits ferroptosis in RBE cells. (A) IC50 values of ferroptosis inducers in MUC1-manipulated RBE cells. (B) Cell viability assay with various cell death pathway inhibitors. (C) Colony formation assay under RSL3 treatment. (D-G) Analysis of ferroptosis features in RBE cells: (D) ferrous ion accumulation, representative images shown at 200× magnification; scale bar = 25 μm, (E) lipid peroxidation levels, (F) ROS production, and (G) mitochondrial morphology. Representative images at different magnifications: 1500× and 5000×; scale bars = 5 μm and 1 μm. (H) Intracellular GSH levels under specified conditions. Data are shown as the means ± SD, the significant level was identified by * *P* < 0.05; ** *P* < 0.01; *** *P* < 0.001; n.s.: no significant.


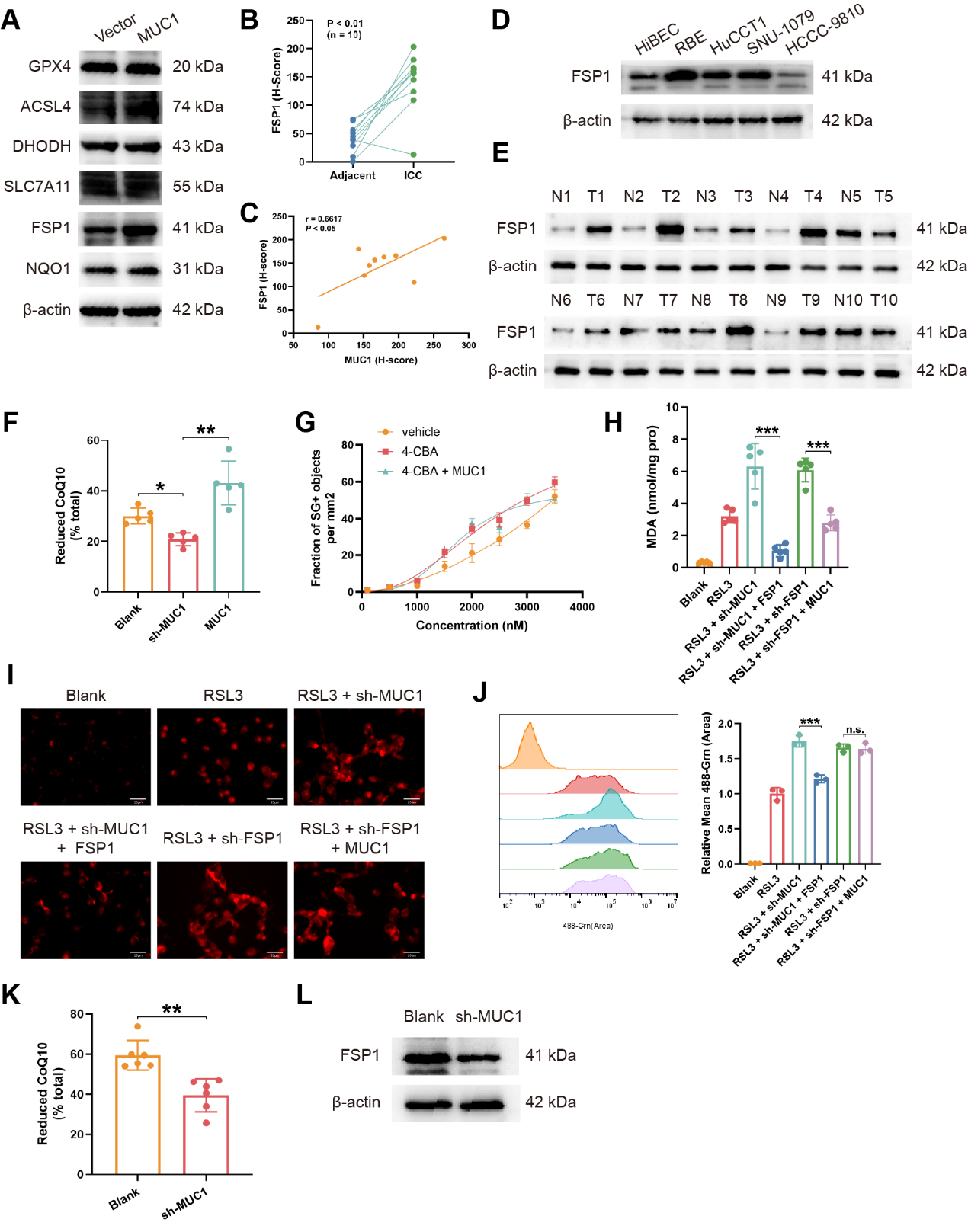


**Figure S3** FSP1 expression analysis and functional validation in ICC. (A) Western blot analysis of ferroptosis-related proteins in RBE cells. (B) IHC H-Score quantification of FSP1 expression in 10 paired ICC and adjacent normal tissue samples. (C) Pearson correlation analysis between MUC1 and FSP1 H-Scores in ICC tissue samples. (D) Quantitative analysis of FSP1 protein expression in ICC cell lines compared to normal biliary epithelial cells using Western blot. (E) Western blot quantification of FSP1 protein levels in 10 paired ICC tissue samples versus adjacent normal tissues. (F) CoQ10 redox status in RBE cells with MUC1 manipulation. (G) SYTOX Green staining in RBE cells treated with RSL3 and 4-CBA. (H-J) Ferroptotic phenotypes in RBE cells with indicated genetic manipulations. Representative images shown at 200× magnification; scale bar = 25 μm. (K) Quantification of reduced CoQ10 ratios in subcutaneous tumors derived from control and sh-MUC1 cells. (L) Western blot analysis of FSP1 protein levels in subcutaneous tumor tissues derived from control and sh-MUC1 cells. Data are shown as the means ± SD, the significant level was identified by * *P* < 0.05; ** *P* < 0.01; *** *P* < 0.001; n.s.: no significant.


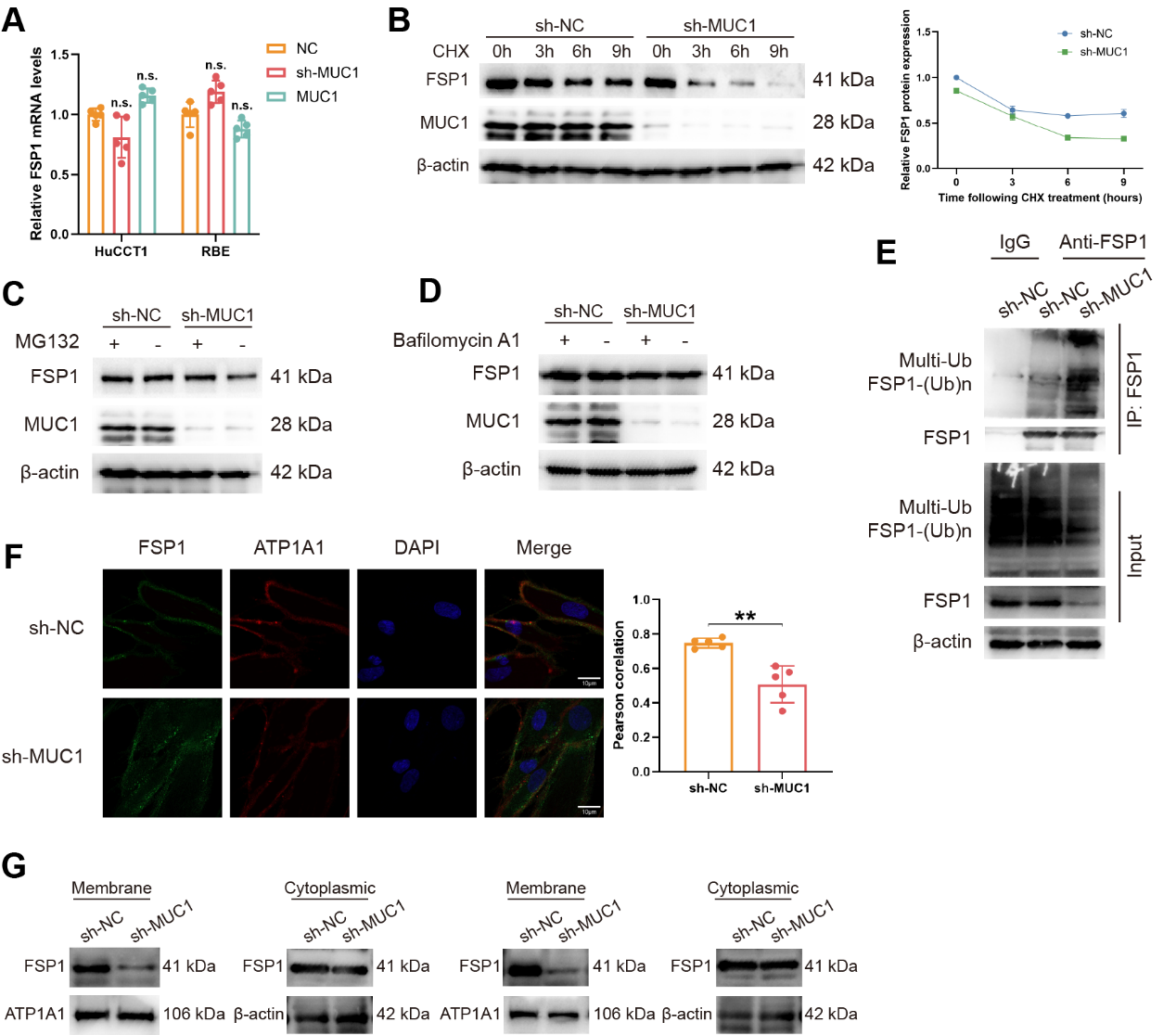


**Figure S4** MUC1 regulates FSP1 at post-translational level. (A) qRT-PCR analysis of FSP1 mRNA expression in ICC cells with MUC1 knockdown or overexpression. (B) Western blot analysis of FSP1 protein degradation in RBE cells treated with CHX (50 μg/ml). (C) Western blot analysis of FSP1 protein levels in RBE cells with MUC1 knockdown treated with or without MG132 (20 μM, 6h). (D) Western blot analysis of FSP1 protein levels in HuCCT1 cells with MUC1 knockdown treated with or without BafA1 (100nM, 6h). (E) Immunoprecipitation assay detecting FSP1 ubiquitination levels in RBE cells. (F) Immunofluorescence co-localization of FSP1 and cell membrane in RBE cells. Representative images shown at 600× magnification; scale bar = 10 μm. (G) Western blot analysis of FSP1 distribution in membrane and cytoplasmic fractions following MUC1 knockdown (left: HuCCT1 cells, right: RBE cells). Data are shown as the means ± SD, the significant level was identified by ** *P* < 0.01; n.s.: no significant.


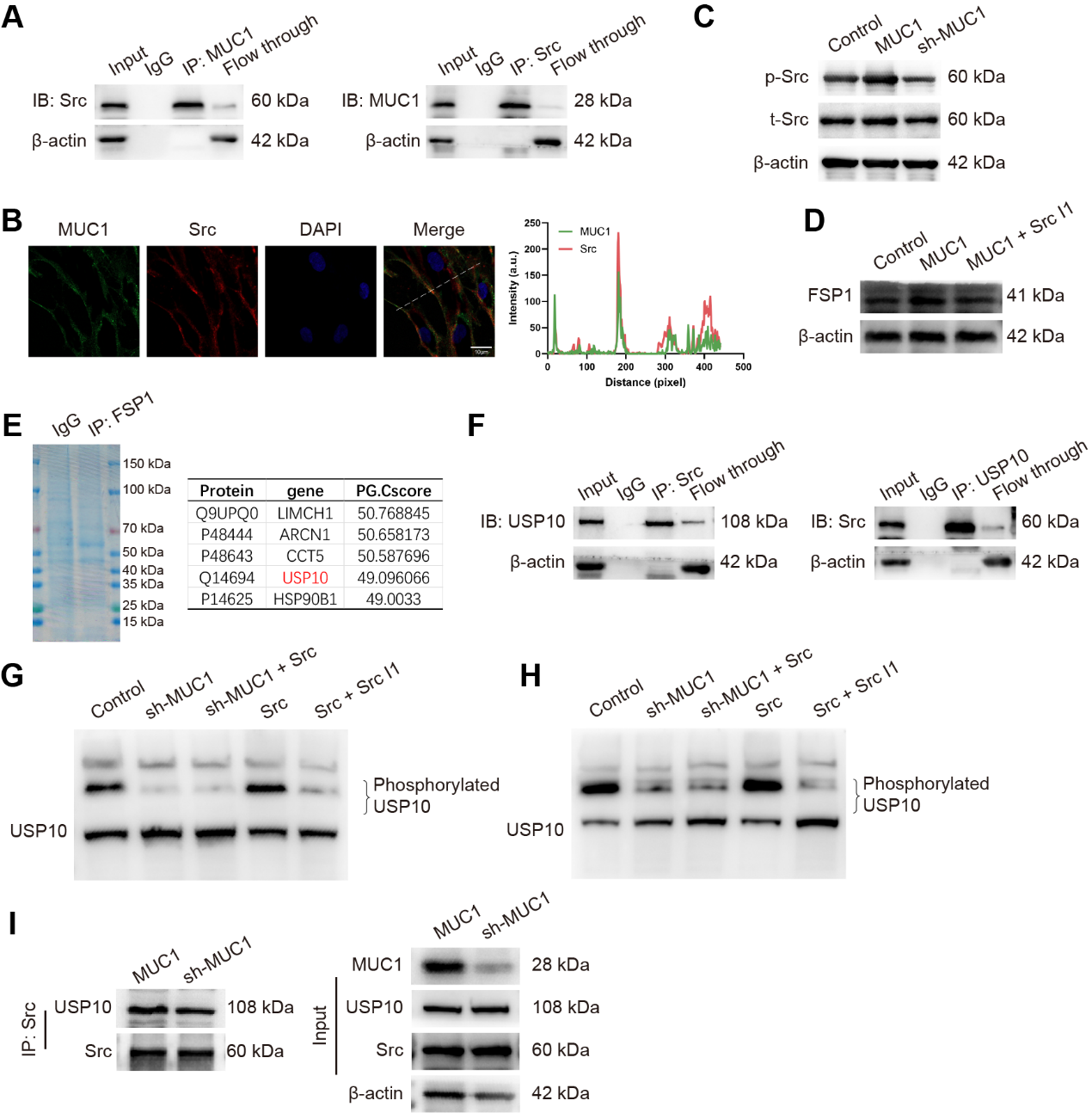


**Figure S5** Additional validation of MUC1-Src-USP10 signaling axis. (A) Co-immunoprecipitation assays in RBE cells. (B) Immunofluorescence co-localization of MUC1 and Src in RBE cells. Representative images shown at 600× magnification; scale bar = 10 μm. (C) Western blot analysis of Src phosphorylation in RBE cells. (D) Western blot analysis of FSP1 in RBE cells treated with Src inhibitors. (E) FSP1 interactome identification in RBE cells: left panel shows Coomassie blue staining of immunoprecipitated proteins (IgG control vs. FSP1-IP); right panel displays tabulated IP-MS results of FSP1-associated proteins. (F) Co-immunoprecipitation assays between Src and USP10 in RBE cells. (G, H) Phos-tag Western blot detection of USP10 phosphorylation in HuCCT1 (left) or RBE (right) cells under different conditions: control, sh-MUC1, sh-MUC1 + Src overexpression, Src overexpression, or Src overexpression + Src inhibitor. (I) Co-immunoprecipitation assays examining Src-USP10 interaction in RBE cells with MUC1 knockdown.


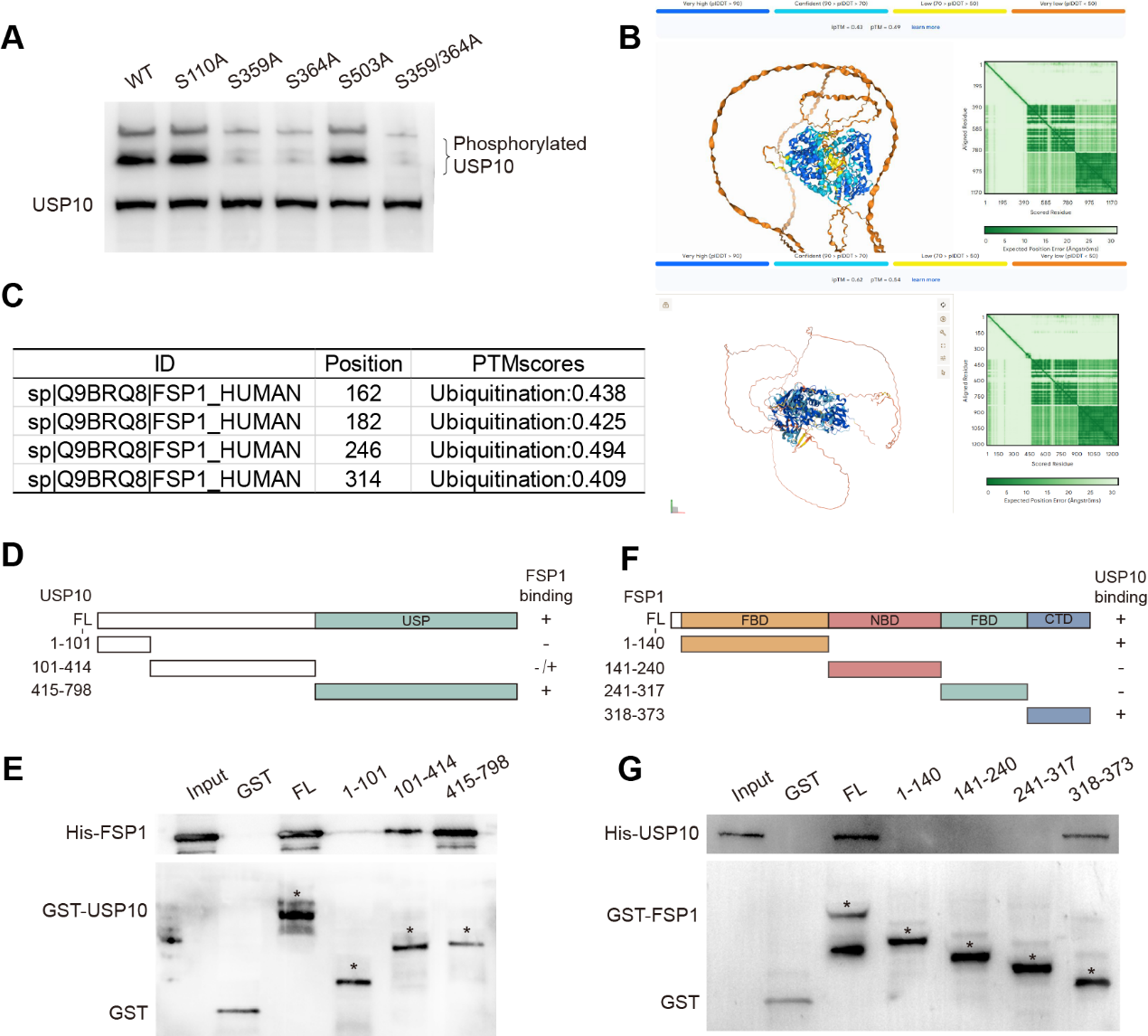


**Figure S6** Detailed characterization of USP10-FSP1 interaction. (A) Phos-tag gel electrophoresis validating Y359 and Y364 as critical Src phosphorylation sites on USP10. (B) Contact probability (ipTM score) comparison between wild-type and phosphorylated USP10 with FSP1 from molecular docking simulations. (C) Musitedeep prediction of ubiquitination sites in FSP1. (D-G) Domain mapping experiments identifying interaction regions between USP10 and FSP1: (D) Schematic of USP10 domain constructs, (E) GST pull down with USP10 truncation mutants, (F) Schematic of FSP1 domain constructs, (G) GST pull down with FSP1 truncation mutants.


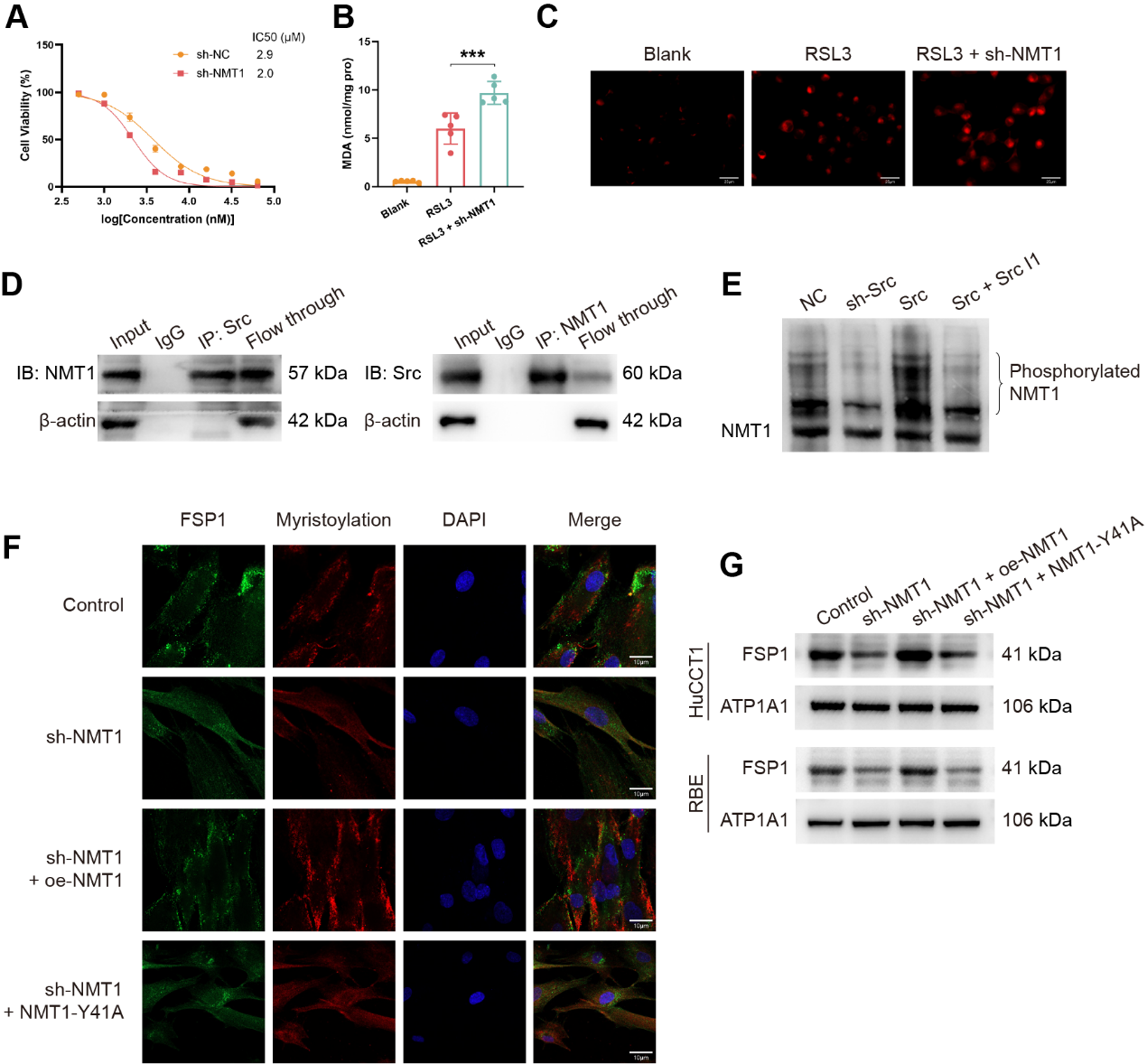


**Figure S7** Further characterization of Src-NMT1-FSP1 signaling axis. (A) RSL3 IC50 determination in HuCCT1 cells following NMT1 knockdown versus negative control. (B) MDA levels in HuCCT1 cells across treatment groups: Blank control, RSL3 treatment, and RSL3 + sh-NMT1 combination. (C) Fe²⁺ staining and quantification in HuCCT1 cells treated with different conditions. Representative images shown at 200× magnification; scale bar = 25 μm. (D) Co-immunoprecipitation assays confirming Src-NMT1 interaction in RBE cells. (E) Phos-tag analysis of NMT1 phosphorylation in RBE cells. (F) Fluorescence microscopy analysis of myristoylation and FSP1 localization in RBE cells. Representative images shown at 600× magnification; scale bar = 10 μm. (G) Western blot analysis of impact of NMT1 phosphorylation site mutations on the distribution of FSP1 in membrane and cytoplasmic fractions. Data are shown as the means ± SD, the significant level was identified by *** *P* < 0.001.

**Supplementary Materials and Methods**

**Cells and reagents**

Human ICC cell lines RBE and HCCC-9810 were obtained from the Cell Bank of the Chinese Academy of Sciences (Shanghai, China), SNU-1079 was purchased from the Korean Cell Line Bank (Seoul, Korea), and HuCCT1 was sourced from Cellverse (Shanghai, China). Immortalized human intrahepatic biliary epithelial cells (HIBEC) were acquired from Otwo Biotech (Guangzhou, China). HEK293 cells were also obtained from the Cell Bank of the Chinese Academy of Sciences (Shanghai, China). RBE, HCCC-9810, SNU-1079, and HuCCT1 cells were maintained in RPMI-1640 medium (Thermo Fisher Scientific), while HIBEC and HEK293 cells were cultured in DMEM medium (Thermo Fisher Scientific), supplemented with 10% fetal bovine serum (Sigma-Aldrich, St. Louis, USA) and 1% penicillin/streptomycin (Beyotime) in a humidified incubator with 5% CO₂ at 37 °C. Cell lines were authenticated by short tandem repeat profiling and tested for mycoplasma contamination every three months.

RSL3, Ferrostatin-1 (Fer-1), Z-VAD-FMK, Necrostatin-1, 3-methyladenine (3-MA), Ac-YVAD-cmk, MG132, Bafilomycin A1 (BafA1), cycloheximide (CHX), and Src inhibitor 1 (Src I1) were purchased from Invivochem (Guangzhou, China). 4-chlorobenzolic acid (4-CBA) was obtained from Sigma-Aldrich (St. Louis, USA).

Antibodies against MUC1 were purchased from Cell Signaling Technology (Danvers, USA). Antibodies targeting FSP1, GPX4, ACSL4, DHODH, SLC7A11, USP10, NMT1, ATP1A1, ubiquitin, and β-actin were obtained from Proteintech (Wuhan, China). Antibodies against Src and phospho-Src (Tyr419) were acquired from Affinity Biosciences (Nanjing, China). Anti-4-hydroxynonenal (4HNE) antibody was purchased from Abcam.

**Patient samples**

Tumor tissues and adjacent non-tumor tissues were collected from 10 ICC patients who underwent surgical resection at the Second Affiliated Hospital of Harbin Medical University. None of the patients received preoperative chemotherapy or radiotherapy. Written informed consent was obtained from all patients, and the study was approved by the Ethics Committee of the Second Affiliated Hospital of Harbin Medical University (approval number: YJSKY2024-245).

**Plasmid construction and gene manipulation**

Human MUC1, Src, USP10, NMT1, and FSP1 cDNAs were cloned into pLVX-Puro, pCDH-CMV-MCS-EF1-Puro, pCMV-Tag2B, or pCMV-Tag3B vectors. Point mutations (USP10 Y359A, Y364A, Y359A/Y364A; NMT1 Y41A; FSP1 K246R) were generated using a site-directed mutagenesis kit (Thermo Fisher Scientific) according to the manufacturer’s instructions. Domain deletion mutants were constructed by PCR amplification of the respective coding regions followed by restriction enzyme digestion and ligation.

For stable overexpression, lentiviral particles were produced by co-transfecting cells with expression vectors and packaging plasmids (psPAX2 and pMD2.G) using Lipofectamine 3000 (Thermo Fisher Scientific). For gene knockdown, shRNA sequences targeting MUC1, USP10, or FSP1 were cloned into the pLKO.1 vector. Lentivirus-containing supernatants were collected 48-72 h post-transfection, filtered, and used to infect target cells. Stable cell lines were selected with puromycin (Thermo Fisher Scientific) at 2 μg/ml for 7 d.

**RNA extraction and quantitative real-time PCR**

Total RNA was extracted using TRIzol reagent (Thermo Fisher Scientific) according to the manufacturer’s protocol. Reverse transcription was performed using a PrimeScript RT Reagent Kit (Sevenbio, Beijing, China). qRT-PCR was conducted on a LightCycler 480 system (Roche) using SYBR Green PCR Master Mix (Sevenbio). Relative expression was calculated using the 2^-ΔΔCt^ method with GAPDH as an internal control. The primers used were as follows: MUC1, Forward: CCTACCATCCTATGAGCGAGTAC, Reverse: GCTGGGTTTGTGTAAGAGAGGC; FSP1, Forward: GACTCCTTCCACCACAATGTGG, Reverse: CAGCACCATCTGGTTCTTCAGG.

**Western blot analysis**

Cells or tissues were lysed in RIPA buffer (Beyotime) supplemented with protease and phosphatase inhibitors (Beyotime). Protein concentration was determined using the BCA Protein Assay Kit (Beyotime). Equal amounts of protein (20-40 μg) were separated by SDS-PAGE and transferred to PVDF membranes (Merck Millipore, Burlington, USA). After blocking with Western Blocking Buffer (Beyotime), membranes were incubated with primary antibodies overnight at 4 °C followed by HRP-conjugated secondary antibodies for 1h at room temperature. Protein bands were visualized using ECL Chemiluminescent Substrate (Biosharp Life Sciences, Hefei, China).

For Phos-tag SDS-PAGE, 50 μM Phos-tag acrylamide (Wako, Osaka, Japan) and 100 μM MnCl_2_ were added to standard SDS-polyacrylamide gels. After electrophoresis, gels were washed three times with transfer buffer containing 10 mM EDTA and then three times with transfer buffer without EDTA before transferring to PVDF membranes.

**Cell viability and colony formation assays**

Cell viability was assessed using the Cell Counting Kit-8 (CCK-8) assay (Dojindo, Kumamoto, Japan). Briefly, cells (5×10^3^ per well) were seeded in 96-well plates and treated as indicated. After treatment, CCK-8 solution was added to each well and incubated for 1-2 h at 37 °C. Absorbance was measured at 450 nm using a microplate reader (Tecan).

For colony formation assays, cells (1×10^3^ per well) were seeded in 6-well plates and treated as indicated. After 10 days, colonies were fixed with 4% paraformaldehyde and stained with 0.1% crystal violet. Colonies containing more than 50 cells were counted under a microscope.

**Cell death analysis**

SYTOX Green staining was used to assess cell death. Cells were treated as indicated and then incubated with 30 nM SYTOX Green (Beyotime) for 20 min at 37 °C. Fluorescence images were captured using a fluorescence microscope (EVOS).

**GSH measurement**

Intracellular glutathione GSH levels were determined using a GSH/GSSG Ratio Detection Assay Kit (Nanjing Jiancheng Bioengineering Institute, Nanjing, China) according to the manufacturer’s protocol. Briefly, cells were lysed and deproteinized, and the supernatant was used for GSH measurement. Fluorescence was measured at excitation/emission wavelengths of 490/520 nm using a microplate reader (Tecan).

**Animal studies**

All animal experiments were approved by the Institutional Animal Care and Use Committee of the Second Affiliated Hospital of Harbin Medical University (approval number: YJSDW2024-230). Female BALB/c nude mice (4-5 weeks old) were purchased from Charles River (Beijing, China) and maintained under specific pathogen-free conditions. For subcutaneous tumor models, HuCCT1 cells (5×10^6^) stably expressing control shRNA or MUC1 shRNA were injected subcutaneously into the right flank of mice (n=6 per group). Tumor volumes were measured every 3 days after Day 9 using calipers and calculated using the formula: volume = (length × width^2^)/2. For drug treatment, mice received RSL3 (10 mg/kg, i.p., every other day) and dasatinib (15 mg/kg, p.o., daily). After 24 days, mice were euthanized, and tumors were harvested, weighed, and processed for histological analysis.

**Cellular fractionation**

Membrane and cytoplasmic fractions were isolated using a Membrane and Cytosol Protein Extraction Kit (Epizyme Biotech, Shanghai, China) according to the manufacturer’s instructions. Protein concentration was determined, and equal amounts of protein from each fraction were analyzed by Western blot. ATP1A1 and β-actin were used as markers for membrane and cytoplasmic fractions, respectively.

**Transmission electron microscopy**

Cells were fixed with 2.5% glutaraldehyde in 0.1 M phosphate buffer (pH 7.4) for 2 h at room temperature, post-fixed with 1% osmium tetroxide for 1 h, dehydrated through a graded ethanol series, and embedded in Epon 812 resin. Ultrathin sections (70 nm) were cut, stained with uranyl acetate and lead citrate, and examined under a transmission electron microscope (Thermo Fisher Scientific) operating at 80 kV.

**Immunohistochemistry**

Formalin-fixed, paraffin-embedded tissue sections (4 μm) were deparaffinized, rehydrated, and subjected to antigen retrieval in citrate buffer (pH 6.0). Endogenous peroxidase activity was blocked with 3% hydrogen peroxide. Sections were incubated with primary antibodies overnight at 4° C, followed by HRP-conjugated secondary antibodies for 1 h at room temperature. Staining was developed using a DAB substrate kit (ZSGB-BIO, Beijing, China) and counterstained with hematoxylin. Images were captured using a light microscope (ZEISS, Oberkochen, Germany).

**GST pull-down assay**

GST-tagged proteins were expressed in E. coli BL21 (DE3) and purified using Glutathione Sepharose 4B beads (Beyotime). His-tagged proteins were expressed and purified using Ni-NTA agarose beads (Beyotime). For pull-down assays, purified His-tagged proteins were incubated with GST-tagged proteins bound to glutathione beads in binding buffer (50 mM Tris-HCl pH 7.4, 150 mM NaCl, 1 mM EDTA, 0.5% NP-40) for 2 h at 4 °C. Beads were washed five times with binding buffer, and bound proteins were eluted with SDS sample buffer and analyzed by Western blot.

**Immunofluorescence staining**

Cells grown on coverslips were fixed with 4% paraformaldehyde (Beyotime) for 15 min, permeabilized with 0.1% Triton X-100 in PBS for 10 min, and blocked with 5% in PBS BSA for 1 h. Cells were incubated with primary antibodies overnight at 4 °C, followed by fluorophore-conjugated secondary antibodies for 1 h at room temperature. Nuclei were counterstained with DAPI (Beyotime).

For visualization of cellular myristoylation, cells were incubated with Click-IT® Myristic Acid Azide (50 μM, Thermo Fisher Scientific) in the dark for 5 h to label N-myristoylated proteins. After labeling, cells were fixed and permeabilized as described above, then reacted with Alexa Fluor® 594 alkyne (Thermo Fisher Scientific) using a Click-iT® Cell Reaction Buffer Kit (Thermo Fisher Scientific) according to the manufacturer’s protocol. For co-localization studies, cells were subsequently blocked with 5% BSA, washed with 0.2% PBST (PBS containing 0.2% Tween-20), and incubated with anti-FSP1 antibody overnight at 4 °C, followed by appropriate secondary antibody incubation. Images were acquired using a confocal microscope (ZEISS) and analyzed using ImageJ software.

**Table S1. Top 200 proteins co-immunoprecipitated with MUC1, ranked by PG. Cscore.**

| **Protein** | **AAs** | **MW (Da)** | **calc.PI** | **Description** | **gene** | **Nr Of Stripped Sequences Identified** | **PG. Cscore** | **PG. Q Value** | **Uniq Count** |
| --- | --- | --- | --- | --- | --- | --- | --- | --- | --- |
| A0AV96 | 593 | 64098.6 | 7.76 | RNA-binding protein 47 | RBM47 | 6 | 59.25273 | 4.07041E-48 | 5 |
| Q13310 | 644 | 70782.3 | 9.75 | Polyadenylate-binding protein 4 | PABPC4 | 8 | 59.25273 | 4.07041E-48 | 7 |
| P30419 | 496 | 56805.9 | 7.91 | Glycylpeptide N-tetradecanoyltransferase 1 | NMT1 | 2 | 56.45948 | 1.63471E-42 | 2 |
| Q9H9T3 | 547 | 62258.4 | 9.12 | Elongator complex protein 3 | ELP3 | 2 | 56.38281 | 2.32837E-42 | 2 |
| Q14694 | 798 | 87133.1 | 4.96 | Ubiquitin carboxyl-terminal hydrolase 10 | USP10 | 8 | 56.096066 | 8.50926E-42 | 8 |
| Q9UBM7 | 475 | 54489 | 8.82 | 7-dehydrocholesterol reductase | DHCR7 | 2 | 55.71625 | 4.69777E-41 | 2 |
| Q01813 | 784 | 85595.4 | 7.6 | ATP-dependent 6-phosphofructokinase, platelet type | PFKP | 14 | 55.66966 | 6.01425E-41 | 14 |
| O76031 | 633 | 69223.2 | 7.64 | ATP-dependent Clp protease ATP-binding subunit clpX-like, mitochondrial | CLPX | 10 | 55.65237 | 6.56099E-41 | 10 |
| P26038 | 577 | 67819.6 | 6.32 | Moesin | MSN | 11 | 55.635517 | 7.14046E-41 | 11 |
| P12931 | 536 | 59834.3 | 7.47 | Proto-oncogene tyrosine-protein kinase Src | SRC | 4 | 54.094116 | 1.12325E-37 | 1 |
| Q99873 | 371 | 42461.3 | 4.99 | Protein arginine N-methyltransferase 1 | PRMT1 | 6 | 54.08213 | 1.19117E-37 | 6 |
| Q14764 | 893 | 99326.2 | 5.15 | Major vault protein | MVP | 10 | 54.080376 | 1.19741E-37 | 10 |
| P52943 | 208 | 22492.6 | 8.85 | Cysteine-rich protein 2 | CRIP2 | 2 | 54.079895 | 1.19741E-37 | 2 |
| Q9NR30 | 783 | 87343.9 | 9.92 | Nucleolar RNA helicase 2 | DDX21 | 9 | 54.078247 | 1.20385E-37 | 7 |
| P07910 | 306 | 33669.7 | 4.69 | Heterogeneous nuclear ribonucleoproteins C1/C2 | HNRNPC | 6 | 54.07347 | 1.23003E-37 | 2 |
| Q9H223 | 541 | 61174.6 | 6.73 | EH domain-containing protein 4 | EHD4 | 14 | 54.064552 | 1.28394E-37 | 12 |
| Q9HAU0 | 1116 | 127463.2 | 7.58 | Pleckstrin homology domain-containing family A member 5 | PLEKHA5 | 2 | 54.0437 | 1.42523E-37 | 2 |
| Q00610 | 1675 | 191613 | 5.42 | Clathrin heavy chain 1 | CLTC | 23 | 54.019535 | 1.60919E-37 | 23 |
| P55884 | 814 | 92480.9 | 4.62 | Eukaryotic translation initiation factor 3 subunit B | EIF3B | 17 | 54.015915 | 1.63441E-37 | 17 |
| P50213 | 366 | 39591.4 | 6.93 | Isocitrate dehydrogenase [NAD] subunit alpha, mitochondrial | IDH3A | 5 | 54.011665 | 1.66542E-37 | 5 |
| Q9BRZ2 | 755 | 81486.9 | 7.78 | E3 ubiquitin-protein ligase TRIM56 | TRIM56 | 11 | 54.00783 | 1.69341E-37 | 11 |
| O75874 | 414 | 46659 | 7 | Isocitrate dehydrogenase [NADP] cytoplasmic | IDH1 | 8 | 54.00533 | 1.71012E-37 | 8 |
| P10809 | 573 | 61054.2 | 5.55 | 60 kDa heat shock protein, mitochondrial | HSPD1 | 16 | 53.99813 | 1.76925E-37 | 16 |
| P13929 | 434 | 46986.5 | 7.84 | Beta-enolase | ENO3 | 6 | 53.997173 | 1.77258E-37 | 6 |
| Q96HE7 | 468 | 54392.1 | 5.41 | ERO1-like protein alpha | ERO1A | 3 | 53.995876 | 1.77904E-37 | 2 |
| P09497 | 229 | 25190.3 | 4.29 | Clathrin light chain B | CLTB | 3 | 53.98661 | 1.86026E-37 | 3 |
| Q15436 | 765 | 86160.1 | 7.07 | Protein transport protein Sec23A | SEC23A | 12 | 53.979862 | 1.92016E-37 | 12 |
| O94826 | 608 | 67454.2 | 7.14 | Mitochondrial import receptor subunit TOM70 | TOMM70 | 3 | 53.971813 | 1.99528E-37 | 3 |
| Q16822 | 640 | 70698.4 | 7.67 | Phosphoenolpyruvate carboxykinase [GTP], mitochondrial | PCK2 | 8 | 53.969517 | 2.01293E-37 | 6 |
| Q96KC8 | 554 | 63882.3 | 8.97 | DnaJ homolog subfamily C member 1 | DNAJC1 | 6 | 53.966774 | 2.03542E-37 | 6 |
| P14868 | 501 | 57135.8 | 6.52 | Aspartate--tRNA ligase, cytoplasmic | DARS1 | 11 | 53.96315 | 2.06751E-37 | 11 |
| Q14257 | 317 | 36876.1 | 4.01 | Reticulocalbin-2 | RCN2 | 2 | 53.96012 | 2.09371E-37 | 2 |
| Q7KZI7 | 788 | 87910 | 10.3 | Serine/threonine-protein kinase MARK2 | MARK2 | 7 | 53.950058 | 2.19829E-37 | 7 |
| Q01780 | 885 | 100830.3 | 8.65 | Exosome complex component 10 | EXOSC10 | 2 | 53.94846 | 2.20987E-37 | 2 |
| P11413 | 515 | 59256.3 | 6.84 | Glucose-6-phosphate 1-dehydrogenase | G6PD | 13 | 53.946087 | 2.23039E-37 | 13 |
| P13646 | 458 | 49587.8 | 4.61 | Keratin, type I cytoskeletal 13 | KRT13 | 3 | 53.94368 | 2.25151E-37 | 1 |
| P12814 | 892 | 103056.7 | 5.07 | Alpha-actinin-1 | ACTN1 | 24 | 53.93973 | 2.29091E-37 | 9 |
| A8MWD9 | 76 | 8544 | 9.38 | Putative small nuclear ribonucleoprotein G-like protein 15 | SNRPGP15 | 1 | 53.931152 | 2.38709E-37 | 1 |
| P13489 | 461 | 49972.7 | 4.44 | Ribonuclease inhibitor | RNH1 | 7 | 53.92367 | 2.47332E-37 | 7 |
| Q9NZR1 | 351 | 39594.7 | 4.93 | Tropomodulin-2 | TMOD2 | 2 | 53.9227 | 2.47849E-37 | 1 |
| P06744 | 558 | 63146.7 | 8.55 | Glucose-6-phosphate isomerase | GPI | 7 | 53.919178 | 2.51638E-37 | 7 |
| P05141 | 298 | 32852 | 10.18 | ADP/ATP translocase 2 | SLC25A5 | 8 | 53.906998 | 2.67092E-37 | 4 |
| Q02878 | 288 | 32727.7 | 11.29 | Large ribosomal subunit protein eL6 | RPL6 | 9 | 53.895626 | 2.82316E-37 | 9 |
| Q08211 | 1270 | 140957.5 | 6.83 | ATP-dependent RNA helicase A | DHX9 | 17 | 53.88975 | 2.90113E-37 | 17 |
| P50579 | 478 | 52891.1 | 5.57 | Methionine aminopeptidase 2 | METAP2 | 5 | 53.88493 | 2.96522E-37 | 5 |
| O75746 | 678 | 74761.2 | 8.56 | Electrogenic aspartate/glutamate antiporter SLC25A12, mitochondrial | SLC25A12 | 9 | 53.877743 | 3.06769E-37 | 4 |
| Q14534 | 574 | 63922.5 | 8.87 | Squalene monooxygenase | SQLE | 3 | 53.870323 | 3.17748E-37 | 3 |
| P23528 | 166 | 18502.3 | 8.29 | Cofilin-1 | CFL1 | 8 | 53.859737 | 3.34499E-37 | 7 |
| P62753 | 249 | 28680.4 | 11.52 | Small ribosomal subunit protein eS6 | RPS6 | 5 | 53.85489 | 3.41937E-37 | 5 |
| P00491 | 289 | 32117.7 | 6.95 | Purine nucleoside phosphorylase | PNP | 5 | 53.840714 | 3.66631E-37 | 5 |
| O00303 | 357 | 37563.5 | 5.12 | Eukaryotic translation initiation factor 3 subunit F | EIF3F | 6 | 53.83391 | 3.78547E-37 | 6 |
| P24534 | 225 | 24763.5 | 4.26 | Elongation factor 1-beta | EEF1B2 | 2 | 53.832348 | 3.80518E-37 | 2 |
| Q53GQ0 | 312 | 34323.9 | 9.79 | Very-long-chain 3-oxoacyl-CoA reductase | HSD17B12 | 6 | 53.823933 | 3.96143E-37 | 6 |
| P17661 | 470 | 53535.3 | 4.94 | Desmin | DES | 7 | 53.818058 | 4.07085E-37 | 7 |
| Q9NZM1 | 2061 | 234706.8 | 6.06 | Myoferlin | MYOF | 11 | 53.782063 | 4.86982E-37 | 11 |
| Q92552 | 414 | 47610.9 | 6.08 | Small ribosomal subunit protein mS27 | MRPS27 | 6 | 53.781895 | 4.86982E-37 | 6 |
| Q8IXH7 | 590 | 66246.1 | 4.74 | Negative elongation factor C/D | NELFCD | 6 | 53.763374 | 5.33783E-37 | 6 |
| Q6NUQ4 | 689 | 77149.8 | 9.52 | Transmembrane protein 214 | TMEM214 | 4 | 53.76036 | 5.40561E-37 | 4 |
| Q7L2E3 | 1194 | 133936.9 | 9.02 | ATP-dependent RNA helicase DHX30 | DHX30 | 14 | 53.754684 | 5.54913E-37 | 14 |
| Q15428 | 464 | 49255.4 | 10.21 | Splicing factor 3A subunit 2 | SF3A2 | 4 | 53.747272 | 5.74712E-37 | 4 |
| P17844 | 614 | 69147.6 | 9.21 | Probable ATP-dependent RNA helicase DDX5 | DDX5 | 19 | 53.74324 | 5.85047E-37 | 14 |
| O43747 | 822 | 91350.5 | 6.78 | AP-1 complex subunit gamma-1 | AP1G1 | 4 | 53.736187 | 6.04814E-37 | 4 |
| Q13561 | 401 | 44230.7 | 4.86 | Dynactin subunit 2 | DCTN2 | 2 | 53.7317 | 6.17121E-37 | 2 |
| P00558 | 417 | 44614.4 | 8.27 | Phosphoglycerate kinase 1 | PGK1 | 15 | 53.729855 | 6.2013E-37 | 13 |
| P31483 | 386 | 42963.1 | 7.85 | Cytotoxic granule associated RNA binding protein TIA1 | TIA1 | 5 | 53.729683 | 6.2013E-37 | 2 |
| P98160 | 4391 | 468826.5 | 6.49 | Basement membrane-specific heparan sulfate proteoglycan core protein | HSPG2 | 4 | 53.728615 | 6.21834E-37 | 4 |
| Q99439 | 309 | 33696.9 | 7.39 | Calponin-2 | CNN2 | 3 | 53.7154 | 6.6335E-37 | 2 |
| P62424 | 266 | 29995.4 | 11.32 | Large ribosomal subunit protein eL8 | RPL7A | 9 | 53.71059 | 6.7798E-37 | 9 |
| P06753 | 285 | 32949.6 | 4.38 | Tropomyosin alpha-3 chain | TPM3 | 6 | 53.708477 | 6.83483E-37 | 5 |
| Q658P3 | 488 | 54600.1 | 8.74 | Metalloreductase STEAP3 | STEAP3 | 3 | 53.70545 | 6.92255E-37 | 3 |
| P55072 | 806 | 89320.9 | 4.89 | Transitional endoplasmic reticulum ATPase | VCP | 24 | 53.702354 | 7.01375E-37 | 24 |
| P36542 | 298 | 32995.7 | 9.71 | ATP synthase subunit gamma, mitochondrial | ATP5F1C | 5 | 53.692753 | 7.34551E-37 | 5 |
| P62910 | 135 | 15859.7 | 11.9 | Large ribosomal subunit protein eL32 | RPL32 | 4 | 53.688633 | 7.47139E-37 | 4 |
| Q14697 | 944 | 106873.1 | 6.06 | Neutral alpha-glucosidase AB | GANAB | 18 | 53.687702 | 7.47139E-37 | 18 |
| Q9UNM6 | 376 | 42945.2 | 5.61 | 26S proteasome non-ATPase regulatory subunit 13 | PSMD13 | 2 | 53.68748 | 7.47139E-37 | 2 |
| P53621 | 1224 | 138344.6 | 7.71 | Coatomer subunit alpha | COPA | 7 | 53.68734 | 7.47139E-37 | 7 |
| Q15434 | 407 | 43958.4 | 9.39 | RNA-binding motif, single-stranded-interacting protein 2 | RBMS2 | 4 | 53.68018 | 7.72829E-37 | 4 |
| Q12797 | 758 | 85862.1 | 4.65 | Aspartyl/asparaginyl beta-hydroxylase | ASPH | 12 | 53.666443 | 8.26585E-37 | 12 |
| Q14247 | 550 | 61585.9 | 5.05 | Src substrate cortactin | CTTN | 12 | 53.663746 | 8.35815E-37 | 12 |
| Q96RN5 | 788 | 86753.3 | 9.97 | Mediator of RNA polymerase II transcription subunit 15 | MED15 | 1 | 53.65241 | 8.83094E-37 | 1 |
| Q05682 | 793 | 93230.5 | 5.35 | Caldesmon | CALD1 | 9 | 53.64307 | 9.23606E-37 | 9 |
| P38606 | 617 | 68303.5 | 5.16 | V-type proton ATPase catalytic subunit A | ATP6V1A | 6 | 53.614197 | 1.06667E-36 | 6 |
| P50502 | 369 | 41331.4 | 4.92 | Hsc70-interacting protein | ST13 | 3 | 53.599373 | 1.14421E-36 |  |
| Q13573 | 536 | 61494 | 10.12 | SNW domain-containing protein 1 | SNW1 | 6 | 53.59935 | 1.14421E-36 | 6 |
| Q07065 | 602 | 66022 | 5.76 | Cytoskeleton-associated protein 4 | CKAP4 | 12 | 53.585873 | 1.22199E-36 | 12 |
| P49411 | 455 | 49874.6 | 7.68 | Elongation factor Tu, mitochondrial | TUFM | 11 | 53.584156 | 1.22952E-36 | 11 |
| O76003 | 335 | 37431.7 | 5.09 | Glutaredoxin-3 | GLRX3 | 6 | 53.5701 | 1.31694E-36 | 6 |
| P51648 | 485 | 54847.4 | 7.99 | Aldehyde dehydrogenase family 3 member A2 | ALDH3A2 | 6 | 53.5693 | 1.31891E-36 | 5 |
| Q96AE4 | 644 | 67560.2 | 7.71 | Far upstream element-binding protein 1 | FUBP1 | 5 | 53.567574 | 1.32713E-36 | 5 |
| Q9BXP5 | 876 | 100665.6 | 5.76 | Serrate RNA effector molecule homolog | SRRT | 9 | 53.566032 | 1.33414E-36 | 9 |
| P21589 | 574 | 63367.3 | 7.04 | 5-nucleotidase | NT5E | 6 | 53.551678 | 1.43112E-36 | 6 |
| Q99536 | 393 | 41920 | 6.25 | Synaptic vesicle membrane protein VAT-1 homolog | VAT1 | 5 | 53.549896 | 1.44045E-36 | 5 |
| Q13740 | 583 | 65101.7 | 6.15 | CD166 antigen | ALCAM | 5 | 53.5417 | 1.49771E-36 | 5 |
| P61604 | 102 | 10931.6 | 9.49 | 10 kDa heat shock protein, mitochondrial | HSPE1 | 6 | 53.536465 | 1.53406E-36 | 6 |
| O60506 | 623 | 69602.3 | 8.88 | Heterogeneous nuclear ribonucleoprotein Q | SYNCRIP | 15 | 53.533802 | 1.55098E-36 | 13 |
| P36776 | 959 | 106488.4 | 6.34 | Lon protease homolog, mitochondrial | LONP1 | 5 | 53.526886 | 1.6022E-36 | 5 |
| O60942 | 597 | 68556.2 | 8.23 | mRNA-capping enzyme | RNGTT | 3 | 53.520275 | 1.65256E-36 | 3 |
| Q02252 | 535 | 57839.3 | 8.69 | Methylmalonate-semialdehyde/malonate-semialdehyde dehydrogenase [acylating], mitochondrial | ALDH6A1 | 3 | 53.511158 | 1.72623E-36 | 3 |
| P33991 | 863 | 96557 | 6.71 | DNA replication licensing factor MCM4 | MCM4 | 12 | 53.508797 | 1.74264E-36 | 12 |
| P15144 | 967 | 109538.7 | 5.14 | Aminopeptidase N | ANPEP | 9 | 53.501057 | 1.80769E-36 | 9 |
| P02533 | 472 | 51561.1 | 4.81 | Keratin, type I cytoskeletal 14 | KRT14 | 23 | 53.483227 | 1.97321E-36 | 9 |
| Q9BQB6 | 163 | 18234.3 | 9.58 | Vitamin K epoxide reductase complex subunit 1 | VKORC1 | 2 | 53.48262 | 1.9744E-36 | 2 |
| P31153 | 395 | 43660.4 | 6.45 | S-adenosylmethionine synthase isoform type-2 | MAT2A | 7 | 53.46959 | 2.10351E-36 | 5 |
| Q07955 | 248 | 27744.3 | 10.77 | Serine/arginine-rich splicing factor 1 | SRSF1 | 8 | 53.463966 | 2.15877E-36 | 8 |
| Q9Y320 | 296 | 34037.3 | 8.91 | Thioredoxin-related transmembrane protein 2 | TMX2 | 2 | 53.461945 | 2.17559E-36 | 2 |
| Q9BQA1 | 342 | 36724 | 4.81 | Methylosome protein WDR77 | WDR77 | 3 | 53.461456 | 2.17566E-36 | 3 |
| O43684 | 328 | 37154.5 | 6.84 | Mitotic checkpoint protein BUB3 | BUB3 | 6 | 53.455467 | 2.23697E-36 | 6 |
| Q9UKV8 | 859 | 97207.5 | 9.54 | Protein argonaute-2 | AGO2 | 10 | 53.451828 | 2.2729E-36 | 10 |
| Q9GZR7 | 859 | 96331 | 9.62 | ATP-dependent RNA helicase DDX24 | DDX24 | 4 | 53.442417 | 2.37763E-36 | 4 |
| Q9UL18 | 857 | 97213.7 | 9.48 | Protein argonaute-1 | AGO1 | 12 | 53.440983 | 2.38912E-36 | 4 |
| P55769 | 128 | 14173.4 | 8.64 | NHP2-like protein 1 | SNU13 | 4 | 53.433575 | 2.47408E-36 | 4 |
| O14980 | 1071 | 123385 | 5.98 | Exportin-1 | XPO1 | 5 | 53.42802 | 2.53823E-36 | 5 |
| Q9UEY8 | 706 | 79154.1 | 6.26 | Gamma-adducin | ADD3 | 8 | 53.42648 | 2.5519E-36 | 8 |
| P41091 | 472 | 51109.1 | 8.54 | Eukaryotic translation initiation factor 2 subunit 3 | EIF2S3 | 13 | 53.418232 | 2.65384E-36 | 2 |
| Q9BS26 | 406 | 46970.7 | 4.9 | Endoplasmic reticulum resident protein 44 | ERP44 | 4 | 53.416637 | 2.66888E-36 | 4 |
| Q9HC38 | 313 | 34793.2 | 5.28 | Glyoxalase domain-containing protein 4 | GLOD4 | 4 | 53.407475 | 2.7831E-36 | 4 |
| Q9NRR5 | 601 | 63852.5 | 4.88 | Ubiquilin-4 | UBQLN4 | 1 | 53.407375 | 2.7831E-36 | 1 |
| O95202 | 739 | 83353.4 | 6.66 | Mitochondrial proton/calcium exchanger protein | LETM1 | 13 | 53.405437 | 2.80375E-36 | 13 |
| P11274 | 1271 | 142818.1 | 7.03 | Breakpoint cluster region protein | BCR | 1 | 53.399426 | 2.88311E-36 | 1 |
| P48735 | 452 | 50908.9 | 8.95 | Isocitrate dehydrogenase [NADP], mitochondrial | IDH2 | 11 | 53.369785 | 3.33899E-36 | 11 |
| P43034 | 410 | 46637.7 | 7.4 | Platelet-activating factor acetylhydrolase IB subunit beta | PAFAH1B1 | 1 | 53.36783 | 3.3641E-36 | 1 |
| P18615 | 380 | 43239.3 | 9.92 | Negative elongation factor E | NELFE | 5 | 53.363007 | 3.4386E-36 | 5 |
| Q99832 | 543 | 59366.1 | 7.73 | T-complex protein 1 subunit eta | CCT7 | 14 | 53.35786 | 3.52048E-36 | 14 |
| P00492 | 218 | 24579.2 | 6.67 | Hypoxanthine-guanine phosphoribosyltransferase | HPRT1 | 6 | 53.357067 | 3.52631E-36 | 6 |
| Q96EY7 | 689 | 78548.9 | 6.38 | Small ribosomal subunit protein mS39 | PTCD3 | 9 | 53.35483 | 3.55787E-36 | 9 |
| P16401 | 226 | 22579.9 | 11.69 | Histone H1.5 | H1-5 | 2 | 53.352238 | 3.59345E-36 | 2 |
| Q96EY1 | 480 | 52488.3 | 9.68 | DnaJ homolog subfamily A member 3, mitochondrial | DNAJA3 | 4 | 53.35193 | 3.59345E-36 | 4 |
| P60891 | 318 | 34833.9 | 6.98 | Ribose-phosphate pyrophosphokinase 1 | PRPS1 | 4 | 53.337765 | 3.84962E-36 | 3 |
| P28074 | 263 | 28480 | 6.94 | Proteasome subunit beta type-5 | PSMB5 | 5 | 53.31329 | 4.34299E-36 | 5 |
| Q96RP9 | 751 | 83470.9 | 7.01 | Elongation factor G, mitochondrial | GFM1 | 5 | 53.29227 | 4.81491E-36 | 5 |
| P53992 | 1094 | 118323.8 | 7.07 | Protein transport protein Sec24C | SEC24C | 11 | 53.282024 | 5.05712E-36 | 11 |
| P50454 | 418 | 46440.1 | 9.12 | Serpin H1 | SERPINH1 | 9 | 53.27831 | 5.1404E-36 | 9 |
| P62913 | 178 | 20252.2 | 10.14 | Large ribosomal subunit protein uL5 | RPL11 | 3 | 53.27732 | 5.15413E-36 | 3 |
| O95573 | 720 | 80419.4 | 8.51 | Fatty acid CoA ligase Acsl3 | ACSL3 | 14 | 53.276424 | 5.16377E-36 | 13 |
| P49189 | 494 | 53801.5 | 5.61 | 4-trimethylaminobutyraldehyde dehydrogenase | ALDH9A1 | 8 | 53.27604 | 5.16377E-36 | 8 |
| P10599 | 105 | 11737.4 | 4.55 | Thioredoxin | TXN | 5 | 53.27101 | 5.28353E-36 | 5 |
| P28482 | 360 | 41389.3 | 6.99 | Mitogen-activated protein kinase 1 | MAPK1 | 4 | 53.261566 | 5.52704E-36 | 3 |
| Q9Y6N5 | 450 | 49960.3 | 9.51 | Sulfide:quinone oxidoreductase, mitochondrial | SQOR | 16 | 53.245094 | 5.98875E-36 | 16 |
| P35555 | 2871 | 312294.7 | 4.55 | Fibrillin-1 | FBN1 | 9 | 53.239777 | 6.13646E-36 | 8 |
| P00403 | 227 | 25564.7 | 4.44 | Cytochrome c oxidase subunit 2 | MT-CO2 | 1 | 53.232674 | 6.33885E-36 | 1 |
| P16118 | 471 | 54680.9 | 6.56 | 6-phosphofructo-2-kinase/fructose-2,6-bisphosphatase 1 | PFKFB1 | 1 | 53.232395 | 6.33885E-36 | 1 |
| P13796 | 627 | 70287.8 | 5.07 | Plastin-2 | LCP1 | 10 | 53.221344 | 6.6842E-36 | 10 |
| P10515 | 647 | 68996 | 7.94 | Dihydrolipoyllysine-residue acetyltransferase component of pyruvate dehydrogenase complex, mitochondrial | DLAT | 3 | 53.21474 | 6.89321E-36 | 3 |
| Q92526 | 530 | 57820.9 | 7.27 | T-complex protein 1 subunit zeta-2 | CCT6B | 1 | 53.20816 | 7.09917E-36 | 1 |
| Q6WKZ4 | 1283 | 137165.8 | 5.09 | Rab11 family-interacting protein 1 | RAB11FIP1 | 4 | 53.207962 | 7.09917E-36 | 4 |
| Q86XP3 | 938 | 102974.5 | 7.01 | ATP-dependent RNA helicase DDX42 | DDX42 | 10 | 53.20555 | 7.16932E-36 | 10 |
| Q15393 | 1217 | 135576.3 | 4.91 | Splicing factor 3B subunit 3 | SF3B3 | 12 | 53.205017 | 7.17258E-36 | 12 |
| P55084 | 474 | 51294 | 9.94 | Trifunctional enzyme subunit beta, mitochondrial | HADHB | 11 | 53.202374 | 7.25195E-36 | 11 |
| P61962 | 342 | 38925.8 | 5.21 | DDB1- and CUL4-associated factor 7 | DCAF7 | 3 | 53.193577 | 7.54501E-36 | 3 |
| P30101 | 505 | 56781.8 | 6.28 | Protein disulfide-isomerase A3 | PDIA3 | 18 | 53.193565 | 7.54501E-36 | 18 |
| Q13616 | 776 | 89677.9 | 8.13 | Cullin-1 | CUL1 | 4 | 53.191532 | 7.60534E-36 | 4 |
| P06733 | 434 | 47168.6 | 7.46 | Alpha-enolase | ENO1 | 10 | 53.189514 | 7.66559E-36 | 10 |
| Q6UB35 | 978 | 105789.1 | 8.15 | Monofunctional C1-tetrahydrofolate synthase, mitochondrial | MTHFD1L | 21 | 53.17654 | 8.16094E-36 | 21 |
| P22735 | 817 | 89786.1 | 5.92 | Protein-glutamine gamma-glutamyltransferase K | TGM1 | 4 | 53.170826 | 8.37886E-36 | 4 |
| Q9UNI6 | 340 | 37687 | 6.84 | Dual specificity protein phosphatase 12 | DUSP12 | 1 | 53.163868 | 8.65615E-36 | 1 |
| P31944 | 242 | 27679.3 | 5.28 | Caspase-14 | CASP14 | 4 | 53.158253 | 8.88286E-36 | 4 |
| O60884 | 412 | 45745.4 | 6.44 | DnaJ homolog subfamily A member 2 | DNAJA2 | 3 | 53.156162 | 8.95658E-36 | 3 |
| Q03135 | 178 | 20471.4 | 5.93 | Caveolin-1 | CAV1 | 3 | 53.155445 | 8.96928E-36 | 2 |
| O94874 | 794 | 89594.4 | 6.77 | E3 UFM1-protein ligase 1 | UFL1 | 14 | 53.147995 | 9.28887E-36 | 14 |
| Q16850 | 509 | 57277.8 | 8.72 | Lanosterol 14-alpha demethylase | CYP51A1 | 5 | 53.144928 | 9.41179E-36 | 5 |
| P35998 | 433 | 48633.4 | 5.65 | 26S proteasome regulatory subunit 7 | PSMC2 | 8 | 53.139156 | 9.66586E-36 | 8 |
| Q9NZ01 | 308 | 36034.1 | 9.79 | Very-long-chain enoyl-CoA reductase | TECR | 6 | 53.118107 | 1.07122E-35 | 6 |
| Q99798 | 780 | 85424.7 | 7.65 | Aconitate hydratase, mitochondrial | ACO2 | 9 | 53.116207 | 1.0791E-35 | 9 |
| O43292 | 621 | 67622.5 | 8.16 | Glycosylphosphatidylinositol anchor attachment 1 protein | GPAA1 | 1 | 53.115486 | 1.08067E-35 | 1 |
| Q8WUF5 | 828 | 89090.1 | 6.8 | RelA-associated inhibitor | PPP1R13L | 2 | 53.106426 | 1.12816E-35 | 2 |
| Q05193 | 864 | 97407.5 | 7.17 | Dynamin-1 | DNM1 | 3 | 53.104248 | 1.13804E-35 | 2 |
| Q00266 | 395 | 43647.6 | 6.24 | S-adenosylmethionine synthase isoform type-1 | MAT1A | 2 | 53.099503 | 1.16279E-35 | 2 |
| P35749 | 1972 | 227337.5 | 5.18 | Myosin-11 | MYH11 | 3 | 53.07768 | 1.29348E-35 | 3 |
| Q9BSJ8 | 1104 | 122855.3 | 5.61 | Extended synaptotagmin-1 | ESYT1 | 4 | 53.068638 | 1.35013E-35 | 4 |
| Q53GS9 | 565 | 65380 | 9.26 | Ubiquitin carboxyl-terminal hydrolase 39 | USP39 | 4 | 53.06588 | 1.36589E-35 | 4 |
| Q9BZF1 | 889 | 101194.8 | 6.95 | Oxysterol-binding protein-related protein 8 | OSBPL8 | 3 | 53.05179 | 1.46194E-35 | 3 |
| O14639 | 778 | 87686.8 | 8.69 | Actin-binding LIM protein 1 | ABLIM1 | 5 | 53.049877 | 1.4728E-35 | 5 |
| O75915 | 188 | 21614.5 | 10.28 | PRA1 family protein 3 | ARL6IP5 | 1 | 53.032898 | 1.5991E-35 | 1 |
| Q6YN16 | 418 | 45394.3 | 8.18 | Hydroxysteroid dehydrogenase-like protein 2 | HSDL2 | 6 | 53.028084 | 1.63437E-35 | 6 |
| P42704 | 1394 | 157903.4 | 6 | Leucine-rich PPR motif-containing protein, mitochondrial | LRPPRC | 29 | 53.021336 | 1.68655E-35 | 29 |
| Q969Q0 | 106 | 12468.8 | 11.35 | Ribosomal protein eL42-like | RPL36AL | 1 | 53.01916 | 1.70131E-35 | 1 |
| P61106 | 215 | 23896.8 | 6.13 | Ras-related protein Rab-14 | RAB14 | 4 | 53.01541 | 1.72968E-35 | 4 |
| Q9Y314 | 301 | 33171.7 | 9.08 | Nitric oxide synthase-interacting protein | NOSIP | 1 | 53.0008 | 1.8559E-35 | 1 |
| P62701 | 263 | 29597.5 | 10.85 | Small ribosomal subunit protein eS4, X isoform | RPS4X | 7 | 52.99523 | 1.9006E-35 | 3 |
| P23246 | 707 | 76149.2 | 9.95 | Splicing factor, proline- and glutamine-rich | SFPQ | 8 | 52.99517 | 1.9006E-35 | 8 |
| P54136 | 660 | 75378.3 | 6.65 | Arginine--tRNA ligase, cytoplasmic | RARS1 | 13 | 52.987537 | 1.96986E-35 | 13 |
| P60900 | 246 | 27399.2 | 6.74 | Proteasome subunit alpha type-6 | PSMA6 | 8 | 52.985493 | 1.98585E-35 | 8 |
| Q9HD20 | 1204 | 132953.5 | 8.2 | Endoplasmic reticulum transmembrane helix translocase | ATP13A1 | 7 | 52.97666 | 2.07047E-35 | 7 |
| P00338 | 332 | 36688.5 | 8.45 | L-lactate dehydrogenase A chain | LDHA | 8 | 52.975082 | 2.08248E-35 | 7 |
| Q16543 | 378 | 44468 | 4.9 | Hsp90 co-chaperone Cdc37 | CDC37 | 4 | 52.973354 | 2.0961E-35 | 4 |
| P35580 | 1976 | 228997.2 | 5.22 | Myosin-10 | MYH10 | 24 | 52.961292 | 2.22067E-35 | 11 |
| O60701 | 494 | 55023.5 | 7.13 | UDP-glucose 6-dehydrogenase | UGDH | 15 | 52.951332 | 2.32823E-35 | 15 |
| Q9Y4K0 | 774 | 86724.3 | 6.35 | Lysyl oxidase homolog 2 | LOXL2 | 1 | 52.943913 | 2.41045E-35 | 1 |
| Q9NVP1 | 670 | 75406.3 | 10.13 | ATP-dependent RNA helicase DDX18 | DDX18 | 6 | 52.929245 | 2.5867E-35 | 6 |
| Q9NX63 | 227 | 26152.1 | 8.45 | MICOS complex subunit MIC19 | CHCHD3 | 2 | 52.92089 | 2.6904E-35 | 2 |
| P15121 | 316 | 35853.1 | 6.99 | Aldo-keto reductase family 1 member B1 | AKR1B1 | 8 | 52.91814 | 2.72176E-35 | 8 |
| Q52LJ0 | 433 | 45547 | 8.93 | Protein FAM98B | FAM98B | 4 | 52.916763 | 2.73484E-35 | 4 |
| O00571 | 662 | 73242.8 | 7.2 | ATP-dependent RNA helicase DDX3X | DDX3X | 23 | 52.913315 | 2.7763E-35 | 5 |
| Q99442 | 399 | 45861.5 | 7.14 | Translocation protein SEC62 | SEC62 | 2 | 52.910843 | 2.80481E-35 | 2 |
| Q92541 | 710 | 80312.8 | 8.49 | RNA polymerase-associated protein RTF1 homolog | RTF1 | 5 | 52.895638 | 3.01771E-35 | 5 |
| P20700 | 586 | 66407.7 | 4.82 | Lamin-B1 | LMNB1 | 13 | 52.891518 | 3.07248E-35 | 13 |
| Q7Z794 | 578 | 61901.1 | 5.78 | Keratin, type II cytoskeletal 1b | KRT77 | 8 | 52.89119 | 3.07248E-35 | 6 |
| O14828 | 347 | 38286.6 | 7.73 | Secretory carrier-associated membrane protein 3 | SCAMP3 | 2 | 52.88792 | 3.1163E-35 | 2 |
| P05787 | 483 | 53703.8 | 5.26 | Keratin, type II cytoskeletal 8 | KRT8 | 20 | 52.885647 | 3.14524E-35 | 19 |
| P51572 | 246 | 27991.4 | 8.89 | B-cell receptor-associated protein 31 | BCAP31 | 3 | 52.879707 | 3.23247E-35 | 3 |

**Table S2. Top 200 proteins co-immunoprecipitated with FSP1, ranked by PG. Cscore.**

| **Protein accession** | **Gene name** | **MW [kDa]** | **Coverage [%]** | **MS/MS Counts** | **Peptides** | **Unique peptides** | **Razor + unique peptides** | **Peptides** | **PG. Cscore** |  |
| --- | --- | --- | --- | --- | --- | --- | --- | --- | --- | --- |
| Q9UPQ0 | LIMCH1 | 121.87 | 46.1 | 35 | 15 | 15 | 15 | 14 | 50.768845 | |
| P48444 | ARCN1 | 57.21 | 60.1 | 53 | 32 | 32 | 32 | 26 | 50.658173 | |
| P48643 | CCT5 | 59.67 | 84.1 | 113 | 52 | 52 | 52 | 40 | 50.587696 | |
| Q14694 | USP10 | 87.133 | 34.1 | 21 | 16 | 15 | 16 | 7 | 49.096066 | |
| P14625 | HSP90B1 | 92.468 | 67.5 | 116 | 58 | 56 | 56 | 51 | 49.0033 | |
| Q86WZ6 | ZNF227 | 92.032 | 4.8 | 3 | 2 | 2 | 2 | 2 | 49.000072 | |
| O14936 | CASK | 105.12 | 7.5 | 4 | 4 | 4 | 4 | 2 | 48.99682 | |
| Q13395 | TARBP1 | 181.67 | 2.5 | 4 | 3 | 3 | 3 | 2 | 48.982395 | |
| Q15382 | RHEB | 20.497 | 29.3 | 5 | 5 | 5 | 5 | 2 | 48.97667 | |
| Q58FF6 | HSP90AB4P | 58.264 | 11.7 | 4 | 9 | 2 | 2 | 9 | 48.971996 | |
| P21741 | MDK | 15.585 | 39.9 | 13 | 6 | 6 | 6 | 6 | 48.96434 | |
| O00566 | MPHOSPH10 | 78.863 | 11.2 | 8 | 5 | 5 | 5 | 5 | 48.960915 | |
| Q5JTH9 | RRP12 | 143.7 | 27.1 | 34 | 25 | 25 | 25 | 23 | 48.954617 | |
| Q99643 | SDHC | 18.61 | 29 | 4 | 3 | 3 | 3 | 2 | 48.94239 | |
| P18085 | ARF4 | 20.511 | 77.2 | 8 | 11 | 6 | 6 | 10 | 48.93865 | |
| Q8IZ81 | ELMOD2 | 34.96 | 18.8 | 5 | 5 | 5 | 5 | 2 | 48.937588 | |
| Q9NQZ2 | UTP3 | 54.557 | 8.8 | 5 | 3 | 3 | 3 | 3 | 48.93375 | |
| P07108 | DBI | 10.044 | 65.5 | 5 | 4 | 4 | 4 | 3 | 48.929142 | |
| O43852 | CALU | 37.106 | 65.4 | 21 | 17 | 17 | 17 | 13 | 48.92847 | |
| P12235 | SLC25A4 | 33.064 | 51.3 | 8 | 21 | 5 | 5 | 18 | 48.92519 | |
| O95777 | LSM8 | 10.403 | 27.1 | 2 | 2 | 2 | 2 | 2 | 48.914646 | |
| Q7LGA3 | HS2ST1 | 41.881 | 25.3 | 7 | 6 | 6 | 6 | 6 | 48.909813 | |
| Q15398 | DLGAP5 | 95.114 | 9.1 | 6 | 5 | 5 | 5 | 2 | 48.8946 | |
| Q9BY67 | CADM1 | 48.509 | 21.9 | 7 | 5 | 5 | 5 | 3 | 48.89288 | |
| Q96G21 | IMP4 | 33.756 | 13.4 | 5 | 4 | 4 | 4 | 3 | 48.891758 | |
| O95071 | UBR5 | 309.35 | 23 | 60 | 46 | 45 | 46 | 38 | 48.87898 | |
| Q7L5D6 | GET4 | 36.504 | 19.6 | 6 | 5 | 5 | 5 | 5 | 48.85403 | |
| Q9UMX5 | NENF | 18.856 | 29.1 | 5 | 4 | 4 | 4 | 3 | 48.846348 | |
| P46379 | BAG6 | 119.41 | 30.4 | 38 | 24 | 24 | 24 | 20 | 48.83778 | |
| Q15392 | DHCR24 | 60.101 | 28.9 | 13 | 12 | 12 | 12 | 7 | 48.837265 | |
| Q9BTV4 | TMEM43 | 44.875 | 11.8 | 6 | 5 | 5 | 5 | 5 | 48.835835 | |
| O94905 | ERLIN2 | 37.839 | 23.3 | 10 | 8 | 7 | 8 | 4 | 48.833347 | |
| P49721 | PSMB2 | 22.836 | 39.8 | 9 | 7 | 7 | 7 | 4 | 48.828712 | |
| Q8NBQ5 | HSD17B11 | 32.963 | 17 | 4 | 3 | 3 | 3 | 3 | 48.828682 | |
| P99999 | CYCS | 11.749 | 49.5 | 12 | 6 | 6 | 6 | 6 | 48.822056 | |
| P40616 | ARL1 | 20.417 | 52.5 | 11 | 8 | 8 | 8 | 5 | 48.820175 | |
| O43747 | AP1G1 | 91.35 | 11.2 | 9 | 8 | 8 | 8 | 6 | 48.807022 | |
| P49720 | PSMB3 | 22.949 | 37.6 | 11 | 6 | 6 | 6 | 5 | 48.80234 | |
| P0DOX8 | -- | 22.83 | 21.3 | 5 | 4 | 4 | 4 | 2 | 48.775364 | |
| O15258 | RER1 | 22.958 | 14.8 | 4 | 2 | 2 | 2 | 2 | 48.769093 | |
| Q15386 | UBE3C | 123.92 | 7.7 | 6 | 6 | 6 | 6 | 3 | 48.759228 | |
| P67812 | SEC11A | 20.625 | 22.9 | 9 | 5 | 5 | 5 | 4 | 48.75561 | |
| P23284 | PPIB | 23.742 | 55.1 | 23 | 13 | 13 | 13 | 9 | 48.754284 | |
| P82970 | HMGN5 | 31.524 | 25.9 | 5 | 4 | 4 | 4 | 3 | 48.751823 | |
| P35659 | DEK | 42.674 | 30.9 | 15 | 11 | 10 | 11 | 10 | 48.736916 | |
| P24390 | KDELR1 | 24.542 | 17.9 | 3 | 3 | 2 | 3 | 3 | 48.732887 | |
| Q9HB40 | SCPEP1 | 50.83 | 13.3 | 5 | 4 | 4 | 4 | 4 | 48.727825 | |
| Q8TCT9 | HM13 | 41.488 | 12.2 | 6 | 4 | 4 | 4 | 4 | 48.726944 | |
| A0MZ66 | SHTN1 | 71.639 | 8.7 | 4 | 4 | 4 | 4 | 2 | 48.726547 | |
| Q9NVU7 | SDAD1 | 79.87 | 15.3 | 16 | 9 | 9 | 9 | 9 | 48.71534 | |
| Q8WVX9 | FAR1 | 59.356 | 11.3 | 6 | 5 | 5 | 5 | 4 | 48.707897 | |
| P49366 | DHPS | 40.97 | 33.3 | 7 | 7 | 7 | 7 | 2 | 48.699436 | |
| Q9H2J4 | PDCL3 | 27.614 | 18.4 | 3 | 3 | 2 | 3 | 2 | 48.69487 | |
| Q9P035 | HACD3 | 43.159 | 30.9 | 12 | 8 | 8 | 8 | 7 | 48.692688 | |
| P11441 | UBL4A | 17.776 | 52.2 | 12 | 7 | 7 | 7 | 6 | 48.688953 | |
| O15260 | SURF4 | 30.394 | 18.6 | 7 | 4 | 4 | 4 | 4 | 48.675068 | |
| Q04323 | UBXN1 | 33.325 | 60.9 | 14 | 10 | 10 | 10 | 5 | 48.672707 | |
| Q8N3U4 | STAG2 | 141.32 | 4.5 | 3 | 3 | 3 | 3 | 2 | 48.66205 | |
| P57088 | TMEM33 | 27.978 | 29.6 | 12 | 8 | 8 | 8 | 7 | 48.651268 | |
| Q9UPN7 | PPP6R1 | 96.723 | 11 | 5 | 4 | 4 | 4 | 2 | 48.64507 | |
| Q7L1Q6 | BZW1 | 48.043 | 36 | 19 | 13 | 12 | 12 | 9 | 48.642574 | |
| P54619 | PRKAG1 | 37.579 | 19.6 | 7 | 6 | 6 | 6 | 5 | 48.64157 | |
| P08123 | COL1A2 | 129.31 | 3.2 | 5 | 4 | 4 | 4 | 3 | 48.640976 | |
| P61803 | DAD1 | 12.497 | 28.3 | 6 | 3 | 3 | 3 | 3 | 48.63854 | |
| P46976 | GYG1 | 39.383 | 12.3 | 4 | 3 | 3 | 3 | 2 | 48.637875 | |
| P49459 | UBE2A | 17.315 | 43.4 | 3 | 3 | 3 | 3 | 2 | 48.625744 | |
| O00264 | PGRMC1 | 21.671 | 59.5 | 17 | 11 | 10 | 11 | 9 | 48.622612 | |
| P56182 | RRP1 | 52.839 | 8 | 4 | 3 | 3 | 3 | 2 | 48.578896 | |
| Q8N7X0 | ADGB | 189.71 | 1 | 3 | 2 | 2 | 2 | 2 | 48.568672 | |
| O95497 | VNN1 | 57.011 | 6.8 | 5 | 3 | 3 | 3 | 2 | 48.56392 | |
| P04080 | CSTB | 11.139 | 70.4 | 9 | 4 | 4 | 4 | 3 | 48.560753 | |
| Q9NV31 | IMP3 | 21.85 | 29.3 | 4 | 4 | 4 | 4 | 4 | 48.559383 | |
| P46977 | STT3A | 80.529 | 14.9 | 12 | 9 | 8 | 8 | 9 | 48.55451 | |
| O14561 | NDUFAB1 | 17.417 | 21.2 | 8 | 4 | 4 | 4 | 4 | 48.5357 | |
| Q9NZM5 | NOP53 | 54.389 | 10.7 | 6 | 4 | 4 | 4 | 4 | 48.5357 | |
| P31689 | DNAJA1 | 44.868 | 71.3 | 33 | 23 | 23 | 23 | 15 | 48.5275 | |
| Q96K17 | BTF3L4 | 17.27 | 57 | 9 | 5 | 5 | 5 | 4 | 48.515434 | |
| P28074 | PSMB5 | 28.48 | 49 | 19 | 11 | 11 | 11 | 9 | 48.502674 | |
| P00395 | MT-CO1 | 57.041 | 7.8 | 5 | 3 | 3 | 3 | 2 | 48.50215 | |
| Q9Y6V7 | DDX49 | 54.226 | 13.3 | 6 | 5 | 5 | 5 | 5 | 48.49612 | |
| Q9H0A0 | NAT10 | 115.73 | 25.5 | 31 | 20 | 20 | 20 | 18 | 48.490177 | |
| P28066 | PSMA5 | 26.411 | 59.8 | 20 | 10 | 10 | 10 | 7 | 48.476353 | |
| P42785 | PRCP | 55.799 | 15.7 | 6 | 5 | 5 | 5 | 4 | 48.474327 | |
| Q96IX5 | ATP5MK | 6.4575 | 44.8 | 6 | 3 | 3 | 3 | 2 | 48.465366 | |
| Q7Z6E9 | RBBP6 | 201.56 | 3.1 | 5 | 4 | 4 | 4 | 4 | 48.452404 | |
| P40926 | MDH2 | 35.503 | 61.5 | 39 | 21 | 21 | 21 | 16 | 48.43728 | |
| Q13404 | UBE2V1 | 16.495 | 57.1 | 12 | 8 | 3 | 8 | 6 | 48.434498 | |
| Q9BZK7 | TBL1XR1 | 55.594 | 10.5 | 4 | 3 | 2 | 3 | 2 | 48.434147 | |
| Q14331 | FRG1 | 29.172 | 14.7 | 5 | 3 | 3 | 3 | 3 | 48.42439 | |
| Q9BQG0 | MYBBP1A | 148.85 | 43.7 | 82 | 45 | 45 | 45 | 40 | 48.416836 | |
| Q7L2H7 | EIF3M | 42.502 | 43.3 | 20 | 12 | 12 | 12 | 8 | 48.411705 | |
| Q8NBS9 | TXNDC5 | 47.628 | 35.6 | 21 | 11 | 11 | 11 | 10 | 48.41142 | |
| Q9UBX3 | SLC25A10 | 31.282 | 31.4 | 12 | 6 | 6 | 6 | 5 | 48.4088 | |
| Q15773 | MLF2 | 28.147 | 41.9 | 10 | 7 | 7 | 7 | 5 |  | |
| Q8TCJ2 | STT3B | 93.673 | 16.5 | 24 | 12 | 11 | 12 | 11 | 48.398926 | |
| Q99805 | TM9SF2 | 75.775 | 14.3 | 11 | 7 | 7 | 7 | 6 | 48.39153 | |
| Q9NZJ7 | MTCH1 | 41.544 | 5.9 | 2 | 2 | 2 | 2 | 2 | 48.386868 | |
| Q9NXS2 | QPCTL | 42.924 | 5.8 | 4 | 2 | 2 | 2 | 2 | 48.386143 | |
| Q6IPR1 | ETFRF1 | 10.864 | 22.2 | 3 | 2 | 2 | 2 | 2 | 48.379726 | |
| Q02978 | SLC25A11 | 34.061 | 50.3 | 30 | 14 | 14 | 14 | 13 | 48.379337 | |
| O43169 | CYB5B | 16.694 | 62 | 13 | 6 | 6 | 6 | 6 | 48.366833 | |
| P23368 | ME2 | 65.443 | 35.1 | 16 | 12 | 12 | 12 | 9 | 48.35007 | |
| Q15269 | PWP2 | 102.45 | 3.2 | 3 | 2 | 2 | 2 | 2 | 48.336483 | |
| P62316 | SNRPD2 | 13.527 | 71.2 | 16 | 11 | 11 | 11 | 8 | 48.33581 | |
| Q8NBX0 | SCCPDH | 47.151 | 14.5 | 6 | 4 | 4 | 4 | 3 | 48.32632 | |
| Q15050 | RRS1 | 41.193 | 32.3 | 10 | 8 | 8 | 8 | 6 | 48.319374 | |
| Q9BVC6 | TMEM109 | 26.21 | 9.5 | 4 | 3 | 3 | 3 | 2 | 48.312782 | |
| Q92621 | NUP205 | 227.92 | 9.1 | 14 | 12 | 11 | 12 | 10 | 48.30722 | |
| Q86UE4 | MTDH | 63.836 | 34.5 | 18 | 14 | 14 | 14 | 7 | 48.298077 | |
| Q96RQ1 | ERGIC2 | 42.548 | 11.4 | 3 | 3 | 3 | 3 | 2 | 48.292114 | |
| Q9H993 | ARMT1 | 51.172 | 27.4 | 7 | 8 | 7 | 8 | 6 | 48.288807 | |
| P56134 | ATP5MF | 10.918 | 42.6 | 7 | 4 | 4 | 4 | 4 | 48.285545 | |
| Q9Y2W2 | WBP11 | 69.997 | 14.7 | 9 | 7 | 7 | 7 | 3 | 48.277374 | |
| Q15070 | OXA1L | 48.547 | 11.5 | 5 | 5 | 5 | 5 | 4 | 48.26582 | |
| P53007 | SLC25A1 | 34.012 | 40.2 | 19 | 12 | 12 | 12 | 11 | 48.262226 | |
| Q99986 | VRK1 | 45.476 | 39.4 | 15 | 9 | 9 | 9 | 8 | 48.259243 | |
| Q9BYD2 | MRPL9 | 30.243 | 38.6 | 14 | 10 | 10 | 10 | 6 | 48.240097 | |
| P24941 | CDK2 | 33.929 | 40.6 | 15 | 10 | 9 | 9 | 7 | 48.235607 | |
| O75494 | SRSF10 | 31.3 | 14.9 | 8 | 5 | 5 | 5 | 3 | 48.233475 | |
| Q9UBM7 | DHCR7 | 54.489 | 11.4 | 6 | 4 | 4 | 4 | 4 | 48.227283 | |
| Q9NXE4 | SMPD4 | 97.809 | 9.4 | 7 | 6 | 6 | 6 | 6 | 48.210415 | |
| P47712 | PLA2G4A | 85.238 | 6.8 | 4 | 3 | 3 | 3 | 2 | 48.200397 | |
| Q15427 | SF3B4 | 44.385 | 15.8 | 5 | 3 | 3 | 3 | 2 | 48.19803 | |
| Q9Y376 | CAB39 | 39.869 | 23.2 | 9 | 7 | 7 | 7 | 6 | 48.19195 | |
| Q13257 | MAD2L1 | 23.51 | 27.3 | 6 | 5 | 5 | 5 | 3 | 48.18725 | |
| P10253 | GAA | 105.32 | 11.4 | 15 | 9 | 9 | 9 | 5 | 48.17155 | |
| P56385 | ATP5ME | 7.9331 | 40.6 | 4 | 3 | 3 | 3 | 3 | 48.154076 | |
| O75190 | DNAJB6 | 36.087 | 11 | 5 | 3 | 3 | 3 | 3 | 48.137215 | |
| Q9Y512 | SAMM50 | 51.976 | 30.7 | 15 | 10 | 10 | 10 | 7 | 48.129986 | |
| P02753 | RBP4 | 23.01 | 23.4 | 11 | 5 | 5 | 5 | 5 | 48.127907 | |
| Q8N0T1 | RBIS | 11.456 | 37 | 4 | 4 | 4 | 4 | 4 | 48.101562 | |
| Q96A26 | FAM162A | 17.342 | 24 | 7 | 5 | 5 | 5 | 4 | 48.100105 | |
| P10619 | CTSA | 54.465 | 14.2 | 6 | 4 | 4 | 4 | 2 | 48.08862 | |
| Q9Y4P1 | ATG4B | 44.294 | 20.4 | 6 | 5 | 5 | 5 | 2 | 48.08052 | |
| P53985 | SLC16A1 | 53.944 | 16.4 | 20 | 9 | 9 | 9 | 9 | 48.079674 | |
| P20338 | RAB4A | 24.389 | 44.5 | 8 | 7 | 7 | 7 | 3 | 48.0795 | |
| Q7Z7F7 | MRPL55 | 15.128 | 30.5 | 6 | 3 | 3 | 3 | 3 | 48.06984 | |
| Q15014 | MORF4L2 | 32.307 | 29.9 | 13 | 8 | 7 | 8 | 6 | 48.068844 | |
| Q9H2W6 | MRPL46 | 31.705 | 47.7 | 14 | 9 | 9 | 9 | 8 | 48.061882 | |
| P28070 | PSMB4 | 29.204 | 42 | 12 | 8 | 8 | 8 | 7 | 48.061176 | |
| Q53GQ0 | HSD17B12 | 34.324 | 39.1 | 15 | 9 | 9 | 9 | 8 | 48.05371 | |
| P04844 | RPN2 | 69.283 | 45.6 | 36 | 20 | 20 | 20 | 14 | 48.022804 | |
| Q9NQP4 | PFDN4 | 15.314 | 28.4 | 5 | 3 | 3 | 3 | 3 | 48.014317 | |
| P04114 | APOB | 515.6 | 1 | 4 | 4 | 3 | 3 | 4 | 48.011543 | |
| O75964 | ATP5MG | 11.428 | 52.4 | 6 | 5 | 5 | 5 | 3 | 48.00994 | |
| Q9NRX1 | PNO1 | 27.924 | 27 | 7 | 5 | 5 | 5 | 4 | 47.98871 | |
| Q16851 | UGP2 | 56.94 | 15 | 7 | 6 | 6 | 6 | 3 | 47.96304 | |
| P61244 | MAX | 18.275 | 13.8 | 2 | 2 | 2 | 2 | 2 | 47.96004 | |
| Q8WTT2 | NOC3L | 92.547 | 8.4 | 6 | 5 | 5 | 5 | 4 | 47.925842 | |
| Q9NSI2 | SLX9 | 25.456 | 20.4 | 6 | 3 | 3 | 3 | 3 | 47.91658 | |
| Q5T9A4 | ATAD3B | 72.572 | 37.3 | 11 | 22 | 8 | 8 | 16 | 47.904873 | |
| P63092 | GNAS | 45.664 | 15.5 | 4 | 4 | 3 | 3 | 3 | 47.890926 | |
| P07099 | EPHX1 | 52.948 | 31 | 18 | 11 | 11 | 11 | 9 | 47.885384 | |
| P07686 | HEXB | 63.137 | 17.1 | 12 | 8 | 8 | 8 | 5 | 47.88408 | |
| Q14165 | MLEC | 32.233 | 16.1 | 6 | 3 | 3 | 3 | 3 | 47.87601 | |
| O75844 | ZMPSTE24 | 54.812 | 20.4 | 10 | 7 | 7 | 7 | 5 | 47.85972 | |
| Q9NP72 | RAB18 | 22.977 | 35 | 7 | 6 | 6 | 6 | 4 | 47.85877 | |
| Q9UNF1 | MAGED2 | 64.953 | 34.3 | 18 | 13 | 12 | 13 | 10 | 47.855103 | |
| Q8N0U8 | VKORC1L1 | 19.835 | 11.9 | 3 | 2 | 2 | 2 | 2 | 47.82663 | |
| Q9BPX3 | NCAPG | 114.33 | 25 | 27 | 19 | 19 | 19 | 15 | 47.824696 | |
| P62899 | RPL31 | 14.463 | 52.8 | 18 | 10 | 10 | 10 | 9 | 47.817112 | |
| Q9Y4C2 | TCAF1 | 102.12 | 9.3 | 10 | 8 | 8 | 8 | 8 | 47.815464 | |
| P28838 | LAP3 | 56.166 | 42.2 | 27 | 16 | 16 | 16 | 15 | 47.812523 | |
| A4D1E9 | GTPBP10 | 42.932 | 20.7 | 8 | 6 | 6 | 6 | 6 | 47.80999 | |
| P31350 | RRM2 | 44.877 | 19.8 | 10 | 6 | 6 | 6 | 4 | 47.801346 | |
| Q9BZE4 | GTPBP4 | 73.964 | 28.5 | 15 | 11 | 11 | 11 | 9 | 47.79768 | |
| O15162 | PLSCR1 | 35.049 | 10.4 | 3 | 2 | 2 | 2 | 2 | 47.79623 | |
| O00488 | ZNF593 | 15.199 | 45.5 | 9 | 4 | 4 | 4 | 4 | 47.78848 | |
| P00491 | PNP | 32.118 | 68.5 | 17 | 15 | 14 | 15 | 8 | 47.784767 | |
| Q9P0I2 | EMC3 | 29.952 | 25.7 | 4 | 4 | 4 | 4 | 4 | 47.754845 | |
| P23258 | TUBG1 | 51.169 | 48.8 | 21 | 14 | 14 | 14 | 8 | 47.748184 | |
| P62495 | ETF1 | 49.03 | 48.5 | 24 | 15 | 15 | 15 | 7 | 47.739513 | |
| Q8N1F7 | NUP93 | 93.487 | 34.4 | 31 | 21 | 21 | 21 | 18 | 47.72247 | |
| Q9BRT6 | LLPH | 15.225 | 38 | 11 | 4 | 4 | 4 | 4 | 47.722054 | |
| P84085 | ARF5 | 20.529 | 81.7 | 15 | 12 | 7 | 7 | 9 | 47.701946 | |
| P60900 | PSMA6 | 27.399 | 45.1 | 16 | 9 | 9 | 9 | 9 | 47.700375 | |
| O60888 | CUTA | 19.116 | 30.7 | 4 | 3 | 3 | 3 | 3 | 47.686306 | |
| Q12996 | CSTF3 | 82.921 | 12.1 | 6 | 5 | 5 | 5 | 3 | 47.68242 | |
| Q6DD88 | ATL3 | 60.541 | 19 | 8 | 5 | 5 | 5 | 5 | 47.678738 | |
| Q5TC12 | ATPAF1 | 36.436 | 9.5 | 3 | 3 | 3 | 3 | 2 | 47.648697 | |
| Q14739 | LBR | 70.702 | 19.3 | 15 | 9 | 9 | 9 | 8 | 47.643436 | |
| P18754 | RCC1 | 44.969 | 55.8 | 22 | 14 | 14 | 14 | 10 | 47.634487 | |
| O43768 | ENSA | 13.389 | 63.6 | 9 | 7 | 5 | 7 | 3 | 47.626663 | |
| Q9H845 | ACAD9 | 68.76 | 25.8 | 18 | 13 | 13 | 13 | 6 | 47.62367 | |
| P62330 | ARF6 | 20.082 | 50.3 | 8 | 5 | 5 | 5 | 3 | 47.61813 | |
| Q8NI27 | THOC2 | 182.77 | 8 | 13 | 10 | 9 | 10 | 8 | 47.615524 | |
| P24928 | POLR2A | 217.17 | 8.7 | 9 | 9 | 8 | 9 | 3 | 47.603706 | |
| Q9Y639 | NPTN | 44.387 | 11.8 | 7 | 3 | 3 | 3 | 3 | 47.602764 | |
| P13667 | PDIA4 | 72.932 | 57.4 | 57 | 34 | 34 | 34 | 27 | 47.58704 | |
| Q9BVS4 | RIOK2 | 63.282 | 8.7 | 4 | 3 | 3 | 3 | 2 | 47.584435 | |
| P30260 | CDC27 | 91.866 | 10.1 | 5 | 4 | 4 | 4 | 3 | 47.578957 | |
| Q9HC07 | TMEM165 | 34.905 | 19.4 | 8 | 4 | 4 | 4 | 3 | 47.552933 | |
| P0DOX5 | -- | 49.328 | 10 | 3 | 3 | 3 | 3 | 3 | 47.533905 | |
| P00505 | GOT2 | 47.517 | 46.3 | 30 | 18 | 18 | 18 | 15 | 47.506954 | |
| O60762 | DPM1 | 29.634 | 43.8 | 15 | 9 | 9 | 9 | 9 | 47.495556 | |
| Q969Z0 | TBRG4 | 70.737 | 16.8 | 10 | 7 | 7 | 7 | 6 | 47.49283 | |
| Q86V81 | ALYREF | 26.888 | 47.9 | 23 | 10 | 9 | 10 | 9 | 47.48574 | |
| Q9UJS0 | SLC25A13 | 74.175 | 43.4 | 41 | 23 | 18 | 23 | 22 | 47.48284 | |
| Q09028 | RBBP4 | 47.655 | 53.9 | 19 | 16 | 10 | 10 | 13 | 47.466183 | |
| Q6P2Q9 | PRPF8 | 273.6 | 36.9 | 102 | 66 | 66 | 66 | 51 | 47.44542 | |
